# Mislabel Identification Using Transfer Learning-Based Ensemble Method

**DOI:** 10.1109/access.2026.3683309

**Published:** 2026-04-14

**Authors:** MD SHARIFUL ISLAM, MIN JUN KIM, PRASHANTA DUTTA

**Affiliations:** 1 School of Mechanical and Materials Engineering, Washington State University, Pullman, WA 99164, USA; 2 Lyle School of Engineering, Southern Methodist University, Dallas, TX 75205, USA

**Keywords:** Adeno-associated virus, mislabels, ensemble model, machine learning, majority filtering, consensus filtering

## Abstract

Accurate labeling of training data is essential for reliable supervised machine learning, particularly in sensitive applications such as virus classification, autonomous driving, precision manufacturing, and medical diagnostics. However, the labeling process is labor-intensive and error-prone. Even widely used datasets such as MNIST and ImageNet contain numerous mislabeled samples. To address this challenge, we developed a transfer learning-based ensemble method that identifies mislabeled data through *majority filtering* and *consensus filtering* using fine-tuned pretrained deep neural networks, including ResNet-50, ResNet-101, VGG-16, EfficientNet, MobileNet, and Inception. Our approach was first validated on the MNIST dataset, where the ensemble detected approximately 751 label inconsistencies, which closely aligns with previously reported estimates of mislabeled samples. Additional experiments with synthetically injected mislabels demonstrated that the method could recover up to 100% of known corrupted labels using majority and consensus voting strategies. The method was then applied to a highly pure adeno-associated virus (AAV) nanopore dataset, where artificial mislabels were introduced for evaluation; the ensemble successfully identified most mislabeled samples and correctly recovered their true labels. Experiments on balanced and unbalanced AAV datasets further showed improved performance on the balanced subset, where all injected mislabels were detected. Compared to classical filtering techniques such as KNN, k-means clustering, and advanced machine learning-based mislabel detection (e.g., DivideMix), *the proposed ensemble method demonstrated superior accuracy, stability, and true-label recovery, establishing it as a strong mislabel detection framework—well-suited for complex, fine-grained datasets such as nanopore signals and other biological measurement data.*

## I. INTRODUCTION

Artificial intelligence (AI) and machine learning (ML) are revolutionizing nearly every sector of modern life, from healthcare and biotechnology to finance to manufacturing. These technologies enable unprecedented levels of automation, pattern recognition, and predictive accuracy, transforming the way we solve complex problems and make data-driven decisions. The rapid advancement in computational power, coupled with the availability of large-scale datasets, has accelerated the development and adoption of intelligent systems across disciplines.

At the core of many ML applications lies supervised learning—an approach where models learn to map inputs to outputs based on labeled examples [1]. Supervised learning has proven particularly effective for classification tasks, in which the goal is to categorize data into predefined classes. These models have demonstrated impressive performance in applications such as image recognition, speech processing, genomics, and medical diagnostics.

Among the most impactful advances in supervised learning are *transfer learning* and *ensemble methods*. Transfer learning enables models to leverage knowledge acquired from large, general-purpose datasets and apply it to domain-specific tasks with limited data. This not only reduces training time and computational costs but also improves generalization in scenarios where labeled data is scarce. Ensemble learning, on the other hand, combines the predictions of multiple models to enhance robustness and reduce overfitting. *When combined, transfer learning and ensemble approaches create powerful frameworks that offer improved accuracy, reliability, and adaptability.*

The integrity of datasets is a cornerstone of supervised machine learning models. Mislabeling, in which the labels in a dataset do not accurately reflect the true class of the corresponding data points, can significantly degrade the performance of supervised learning models. This labeling error can result from various sources, including, but not limited to, data-entry errors, subjectivity, and insufficient information about the object [2].

Proper handling of mislabeled data is essential for machine learning models, as it directly affects the validity of classification results, reproducibility, and generalizability. Mislabeled data can lead to erroneous conclusions, wasted resources, and potential setbacks in critical fields such as autonomous systems, healthcare, and bioinformatics. For instance, the elimination of mislabels in autonomous systems is crucial for ensuring safety, reliability, and performance. These systems—such as self-driving cars, drones, and robotic assistants—rely heavily on machine learning models trained on labeled data. When the labels are incorrect, the models can learn misleading patterns, resulting in poor decisions or unsafe behavior [3]. Moreover, mislabeled data can slow down training, confuse model validation, and reduce trust in the system’s outputs [4]. In medical diagnostics, any mislabels in datasets used for disease identification or treatment planning can lead to incorrect predictions, compromising patient safety and health [5], [6], [7]. For instance, mislabeled imaging data can result in false negatives or positives, impacting clinical decisions [8], [9]. *Addressing mislabeled data ensures that machine learning models used in healthcare deliver reliable predictions, thereby advancing precision medicine and reducing the risks associated with diagnostic errors.*

This work presents a method for identifying mislabels in image-based datasets to address the aforementioned concerns. We specifically apply this approach to an AAV nanopore dataset, where accurate labeling is essential for downstream viral classification and analysis. Our specific contributions are:

- Developing a transfer learning-based heterogeneous ensemble for mislabel detection using majority and consensus filtering.
- Demonstrating its effectiveness on the MNIST dataset with both natural and injected label noise.
- Applying the method to the AAV nanopore dataset with controlled mislabel insertion.
- Providing an analysis of voting strategies, model confidence thresholds, classifier combinations, and data-balance effects.
- Presenting a practical mislabel-detection workflow suitable for small experimental datasets.

The remainder of this paper is organized as follows: Section II provides a detailed review of related literature; Section III describes the methodology and the design of the ensemble framework; Section IV presents the validation results and analysis using both MNIST and AAV datasets; and Section V concludes with key findings and directions for future work.

## II. LITERATURE REVIEW

Mislabeled class noises are generally handled with two categories of models: *noise-tolerant robust models and models requiring processed, cleaned data* [10]. In the first category, the models are designed in such a way that they become noise-tolerant. This is usually done by using carefully crafted loss functions or regularization terms [11]. In some models, boosting-based methods like Adaboost and Bagging are used to handle mislabeled data [12], [13].

Recent advances in noisy-label learning have introduced several powerful, robust training strategies. For example, Co-teaching trains two networks simultaneously, where each network selects small-loss samples and teaches them to the other, reducing the effect of noisy labels during training [14]. Similarly, DivideMix combines a Gaussian mixture model (GMM) based separation of clean and noisy samples with semi-supervised consistency learning [15], achieving strong performance on large computer-vision datasets [16]. Another class of methods, such as Negative Learning, uses complementary (negative) labels to reduce the impact of incorrect annotations by explicitly discouraging the model from predicting the given noisy label [17].

Although these noise-tolerant models are effective on large benchmark datasets, they often rely on assumptions such as clear separation in loss distributions, large sample sizes, and noticeable interclass differences. When these conditions are not met, their ability to distinguish clean from noisy labels can degrade significantly. This limitation appears in many real-world scenarios where datasets are either small or contain fine-grained class differences. The AAV nanopore dataset used in this work is one such example, as it is limited in size and exhibits subtle variations between classes, making robust training methods less practical for reliable mislabel identification.

The second category relies on data filtering and preprocessing to address mislabeled classes. To tackle the challenge of mislabeled data using data filtering and preprocessing, current studies mostly focus on two types of techniques: k-nearest neighbor (KNN) models and ensemble learning-based models [18]. The idea of the KNN model is to compare the labels of neighboring points and check for any inconsistencies [19]. This model suffers from high computation time for prediction. On the other hand, ensemble learning is widely used for the identification of mislabels [20], [21], [22], [23], [24], [25]. In ensemble learning, multiple independent learning algorithms are combined to mitigate the limitations of any individual learning algorithm. Usually, a majority filtering (MF) or consensus filtering (CF) method is used to vote among the predictions made by all the classifiers [22].

It is difficult to obtain large amounts of labeled data in many practical applications. For this reason, in some studies, unlabeled data are also used alongside labeled data to aid in mislabel identification [21], [26]. These unlabeled data, which are cheaper to produce, can still capture the underlying structure and distribution of a dataset. Even with a large dataset, the choice of ensemble plays a crucial role in mislabel identification. Hasan and Chu [10] developed a heterogeneous ensemble by combining k-means clustering and classifier calibration. Classifier calibration helps to adjust the raw confidence scores of individual classifiers to better reflect the true likelihood. Maryam et al. [27] employed the bootstrap ensemble technique to identify classification noise. With the advantage of bootstrap sampling, this procedure is effective for high levels of class noise.

It is evident that the choice of the base learner classifier is very important for a particular set of data. Sluban and Nada [28] showed that a higher diversity of ensembles is most effective for consensus-based ensemble models, while majority filtering approaches often benefit from less diversity or more homogeneous feature representations. Although ensemble methods for mislabel identification have been widely explored using traditional machine-learning techniques—such as k-nearest neighbor, naïve Bayes, decision trees, and support vector machines [21], [26], the use of pretrained deep neural networks in filtering-based mislabel identification remains relatively limited. For example, Zhang [29] proposed an improved method for identifying mislabeled samples in MNIST and CIFAR-10 by training multiple convolutional neural network (CNN) models on different subsets of the dataset; however, their filtering strategy relied on repeated training of the same architecture. Such homogeneous ensembles may fail to capture complementary feature representations arising from architectural diversity. *In contrast, heterogeneous ensembles built from transfer learning based pretrained models can leverage richer and more generalizable features learned from large datasets, offering faster training and improved robustness even with limited data [30], [31], [32].*

In this work, we utilized fine-tuning based transfer learning models forming ensembles to detect mislabels in image-type datasets. The transfer learning has proven to be very useful in many machine-learning applications [33], [34], [35]. The concept of transfer learning comes from solving one problem by implementing the knowledge gathered from another related application [36]. Many highly effective pre-trained classifiers have been developed based on the ImageNet dataset, which are then successfully used for other image-based datasets [37]. For instance, Bansal et al. [38] used transfer learning for classification in the Caltech-101 dataset; Vallabhajosyula et al. [31] implemented transfer learning based ensembles to identify plant diseases. Transfer learning based machine learning models were also employed to classify AAVs with promising accuracy in distinguishing different viral classes [39]. Motivated by the success of these transfer learning-based models, we’ve formed ensembles using popular deep CNN models pre-trained on ImageNet data for our problem. *Unlike traditional ensembles, which are composed of shallow classifiers, the diverse, heterogeneous, and powerful feature extractors in these pretrained models enable more reliable detection of subtle inconsistencies in small, fine-grained datasets.* The proposed method is applied to an AAV nanopore dataset—an area where mislabeled data have significant scientific and clinical implications—and demonstrates strong performance even when mislabels are artificially introduced into highly pure data. *This unique combination of deep transfer learning, ensemble-based filtering, and application to AAV data obtained from nanopore imaging constitutes the primary contribution of this work.*

## III. METHODOLOGY

**FIGURE 1.**
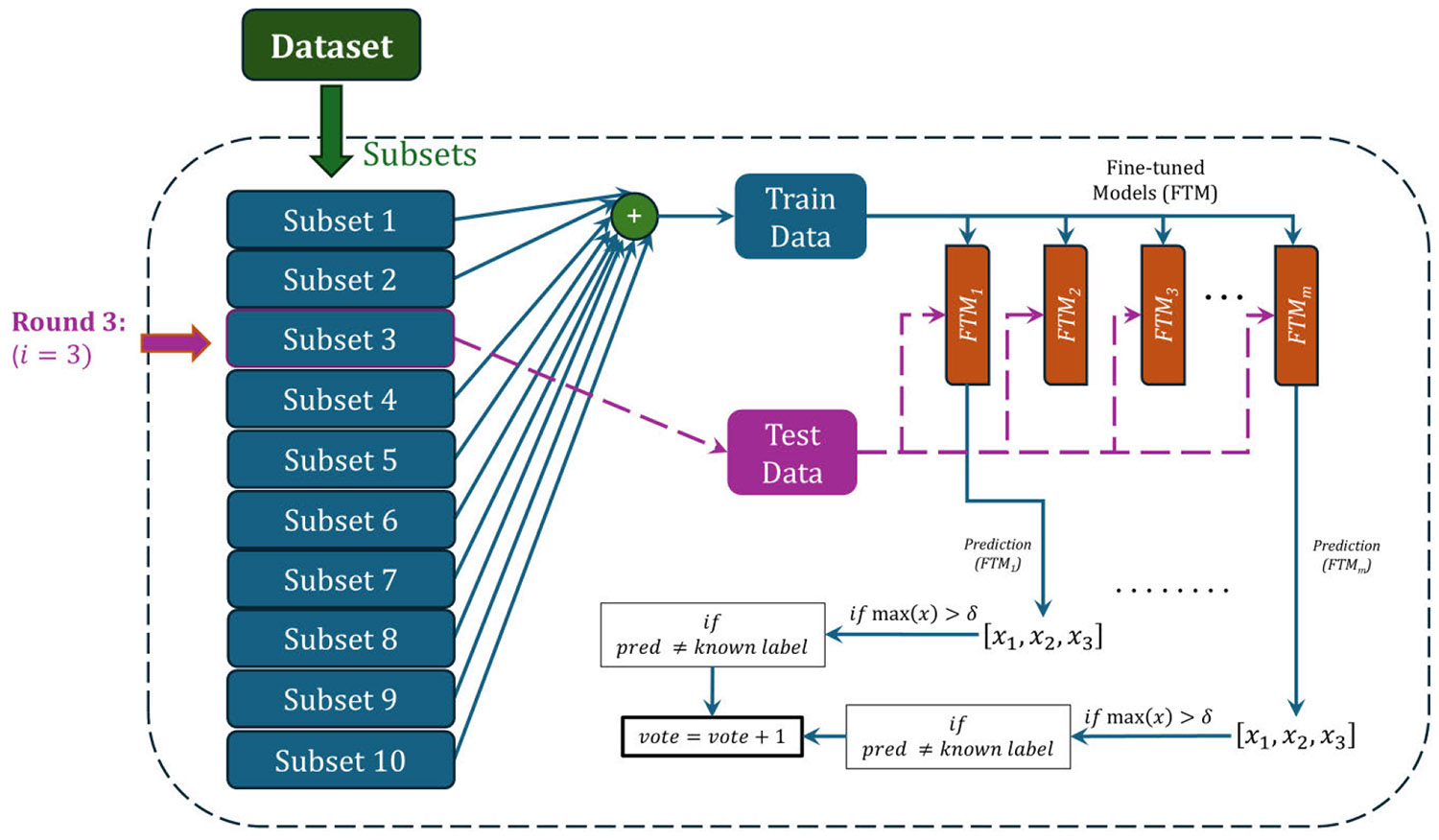
A graphical representation of the filtering algorithms for a specified Softmax confidence threshold value (*0δ*). This process follows a cross-validation training strategy where the whole dataset is divided into n subsets. In each round, one subset is reserved for testing while the remaining subsets are used for training all the fine-tuned models (FTM). Various well-known pretrained deep convolutional neural network models are used to develop fine-tuned classifiers. For the classification problem presented here, $x_{1}$, $x_{2}$ and $x_{3}$ are the three elements of the Softmax prediction vector obtained from fine-tuned classifiers for data with three classes (AAV nanopore dataset). The counting of votes in each round is shown here, while the voting method will decide the minimum number of votes required to identify a mislabel. The round number will determine the test set, and the remaining data will be used in the training set. Here, the data is divided into 10 subsets (n = 10), and the setup is shown for a single round, i = 3.

The ensemble learning model used to identify the mislabels in the dataset is shown in Figure 1. In our ensemble model, we used five fine-tuned classifiers. These classifiers are built based on the widely used deep CNN: ResNet-50 [40], [41], VGG-16 [42], EfficientNet [43], Inception [44], and ResNet-101 [45]. In one case, MobileNet V2 [46] was added to the ensemble, replacing another model. Previous studies have demonstrated that these base classifiers are an excellent choice to develop fine-tuned models for a variety of image-based classification problems. While it is tempting to develop deep CNN-based classifiers from scratch, the utilization of these existing classifiers can reduce the model training time significantly. However, these pre-trained models were developed and trained on the ‘ImageNet’ dataset [47]. Thus, to fit our dataset into these models, changes were made to the last three layers, as discussed in Section III-B.

*To detect mislabeled samples in the dataset, the fine-tuned models* (*FTM* : 1, 2, 3, . . . , *m*) *are trained using a* ***cross-validation training*** *strategy, which is different than the traditional k-fold cross-validation. In standard k-fold cross-validation (CV), a single model architecture is trained repeatedly across k different train/validation splits to estimate performance. However, in our approach, we use a similar folding strategy but for a different purpose: each held-out fold is evaluated by the ensemble to detect mislabeled samples. Thus, while k-fold CV trains the same model multiple times to measure accuracy, our method aggregates predictions across folds to identify label inconsistencies.* In our method, the dataset is divided into *n* subsets. For each cross-validation round, one subset is held out as the testing set, while the remaining (*n* − 1) subsets are used for training all fine-tuned models. Mislabels are then identified only within the held-out subset, ensuring that the model has never seen these samples during training. For example, in the third round of cross-validation (Fig. 1), subset #3 is used for testing while all other subsets are used for training. By repeating this procedure *n* times, every sample in the dataset is evaluated exactly once as unseen test data.

After each cross-validation round, each fine-tuned model computes a probability distribution over the images in the held-out subset. If the predicted probability for a class exceeds a predefined confidence threshold, that model becomes eligible to vote in the subsequent filtering step. It is important to emphasize that each cross-validation round is performed independently: model weights are reset to their original pretrained values before the next round, and no knowledge is carried over between rounds. Additionally, we do not compute or report classification accuracy for these cross-validation trained models, as the purpose of this step is strictly to identify mislabels rather than to evaluate classification performance.

### A. MODEL ARCHITECTURE OF PRE-TRAINED BASE CLASSIFIERS

A maximum of five pre-trained models were used in the ensemble process (out of six selected pre-trained models presented in Table 1), each with a distinct architecture that contributes uniquely to feature extraction. The key architectural features and top-1 accuracy on the ImageNet dataset for each model are summarized in Table 1. Additionally, the number of trainable parameters for ImageNet training is included. *These models were selected for their complementary architectures, their common use in noisy-label research, and their ability to produce classifier diversity.*

Notably, although VGG-16 has the fewest layers among the models, it contains a very large number of trainable parameters. This is primarily due to its use of fully connected layers, which significantly increases the parameter count. In contrast, EfficientNet-B0 has the fewest trainable parameters, owing to its use of mobile inverted bottleneck convolution blocks, squeeze-and-excitation attention mechanisms, skip connections, and the avoidance of large fully connected layers. Similarly, Inception V3 achieves high accuracy with relatively few parameters by leveraging parallel processing paths within its inception modules and using 1 × 1 convolutions for efficient dimensionality reduction.

**FIGURE 2.**
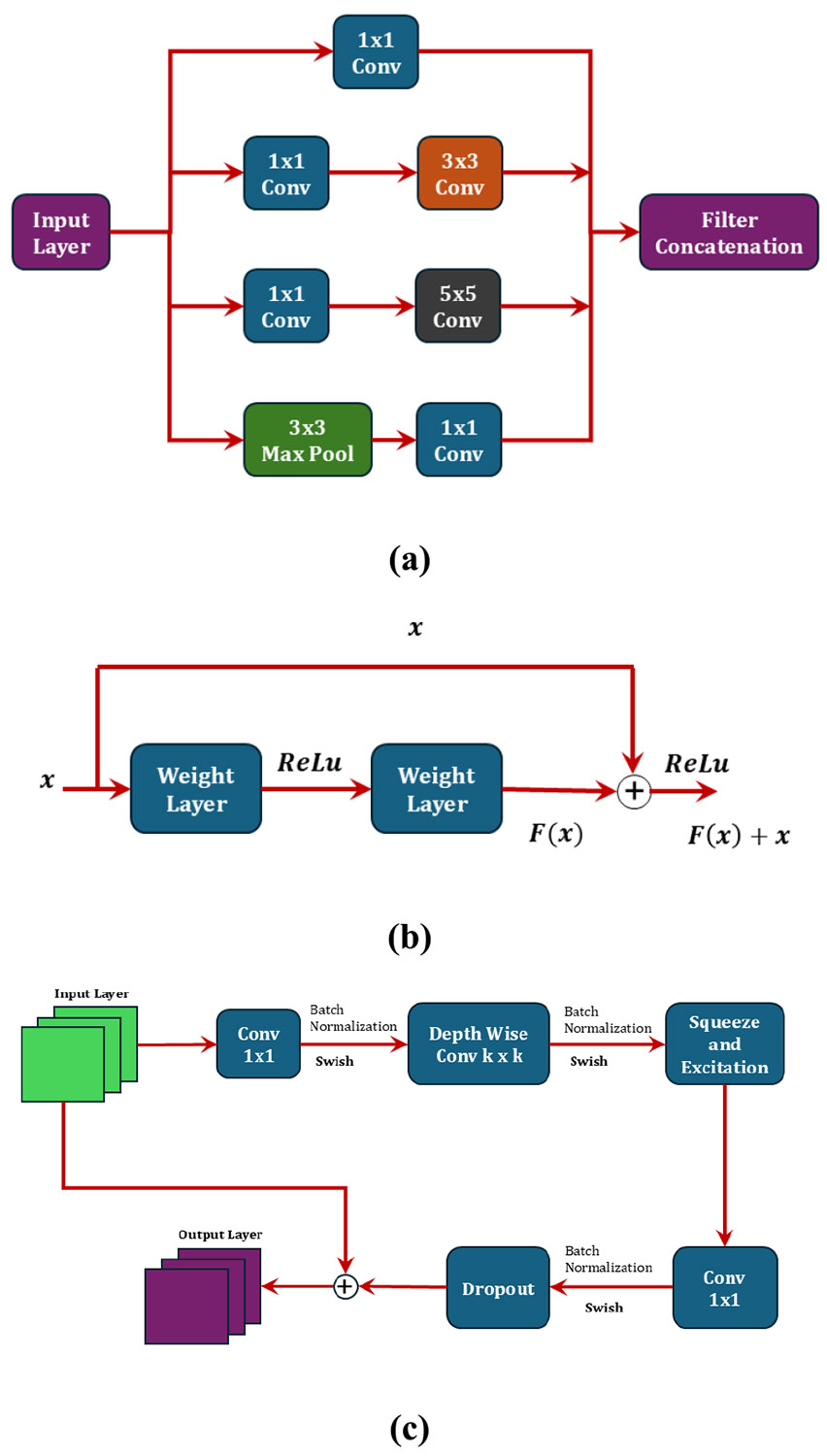
The unique building blocks: (a) Inception block used in Google Inception V3 model; (b) Residual block used in ResNet-50 and ResNet-101 models; (c) Mobile inverted bottleneck convolution (MBConv) block used in EfficientNet B0 model. MBConv blocks are also present in the MobileNet V2 model as a linear bottleneck layer. The inception block splits the input into multiple convolutional processes with different kernel sizes, and the outputs are then concatenated. The residual block uses a skip connection from the input to the output, which bypasses the weight layers. The MBConv block, along with the convolution and Squeeze-and-Excitation, also uses a skip connection directly to the output.

Each model mentioned here has unique core feature blocks. These blocks are shown graphically in Fig. 2. The Inception V3 model uses the Inception module, which splits the input into multiple parallel branches to enable multi-scale feature extraction. In contrast, both ResNet-50 and ResNet-101 are built on the residual block shown in Fig. 2(b), which helps address the vanishing gradient problem and enables effective training of very deep networks. EfficientNet, on the other hand, incorporates the mobile inverted bottleneck convolution block, illustrated in Fig. 2(c), which contributes to its parameter efficiency and strong performance.

### B. MODIFICATION OF THE PRE-TRAINED MODELS

Since the number of classes in our case is different from the ImageNet dataset, appropriate modifications were made to the last few layers of the model.

**FIGURE 3.**
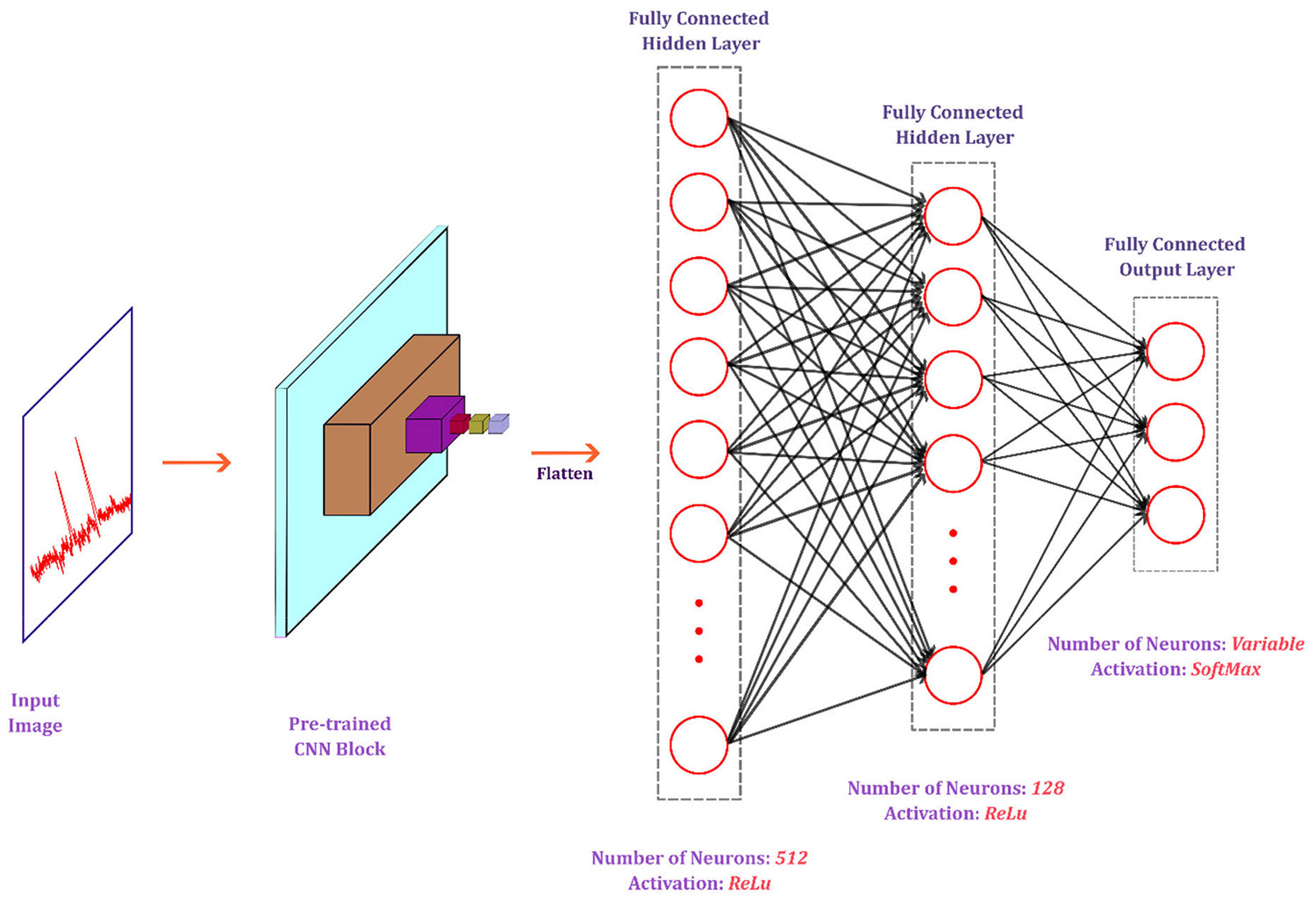
Modifications in the pre-trained models were performed in the fully connected (FC) layers. To match the number of classes, the number of neurons was adjusted. The convolution layers before the FC layers remained the same, and the weights and biases were imported from ImageNet training. The number of neurons and activation functions is also shown for the FC layers.

Figure 3 presents the changes in the last three fully connected layers and the final classifier layer. The number of neurons was initially high and then decreased to match the number of classes in the targeted dataset. ‘ReLu’ activation function was used for the intermediate layers, and ‘Softmax’ [48] was used for the final (output) layer. This Softmax activation function outputs a probability distribution vector over all classes.


(1) $$P\;(y_{i})=\text{Softmax}\;(z_{i})=\frac{e^{z_{i}}}{\sum_{j=1}^{K}e^{z_{j}}}$$

Here, K is the total number of classes, $z_{i}$ is the raw output from the previous layer, and $\sum_{j=1}^{K}e^{z_{j}}$ term indicates the sum of the exponential terms for all classes. Softmax basically transforms the raw output values to the likelihood of each class being the accurate/correct one. This also ensures the sum of probabilities of all classes is one. The predicted label is determined using:

(2) $${\hat{y}}_{i}=\text{arg}\;\max_{i}\;(P(y_{i}))$$

In all models, a loss term is used, which is minimized to find the optimized set of weights and biases in the modified layers. For multi-class classification problems, ‘categorical cross-entropy’ is the most popular loss function. In the present study, this loss term is calculated as

(3) $$L\;(y,\hat{y})=-\sum_{i=1}^{K}y_{i}\;\log\;({\hat{y}}_{i})$$

Here, $y_{i}$ is the true label and ${\hat{y}}_{i}$ is the predicted output for $i^{th}$ class. Before that, all labels are preprocessed to convert into one-hot encoding, which converts categorical data into a binary vector format. After defining the model architecture, all models are combined into an ensemble using the filtering algorithms discussed below.

### C. FILTERING ALGORITHM

To incorporate the ensemble with all the fine-trained classifiers, Brodley’s majority filtering (MF) and consensus filtering (CF) ideas were used [2]. The general concept of MF and CF is as follows: instead of relying upon a single classifier, a set of fine-tuned classifiers is used to detect mislabeled instances depending on their votes.

A sample or instance is tagged as a mislabel if a certain number of the *m* base-level classifiers cannot classify it correctly. In the case of MF, an instance is tagged as mislabeled if more than half of the m base-level classifiers predict it incorrectly, whereas in CF, all the classifiers should classify it incorrectly. Here, incorrect prediction means failure to identify the known or assigned label of a sample.

**FIGURE 4.**
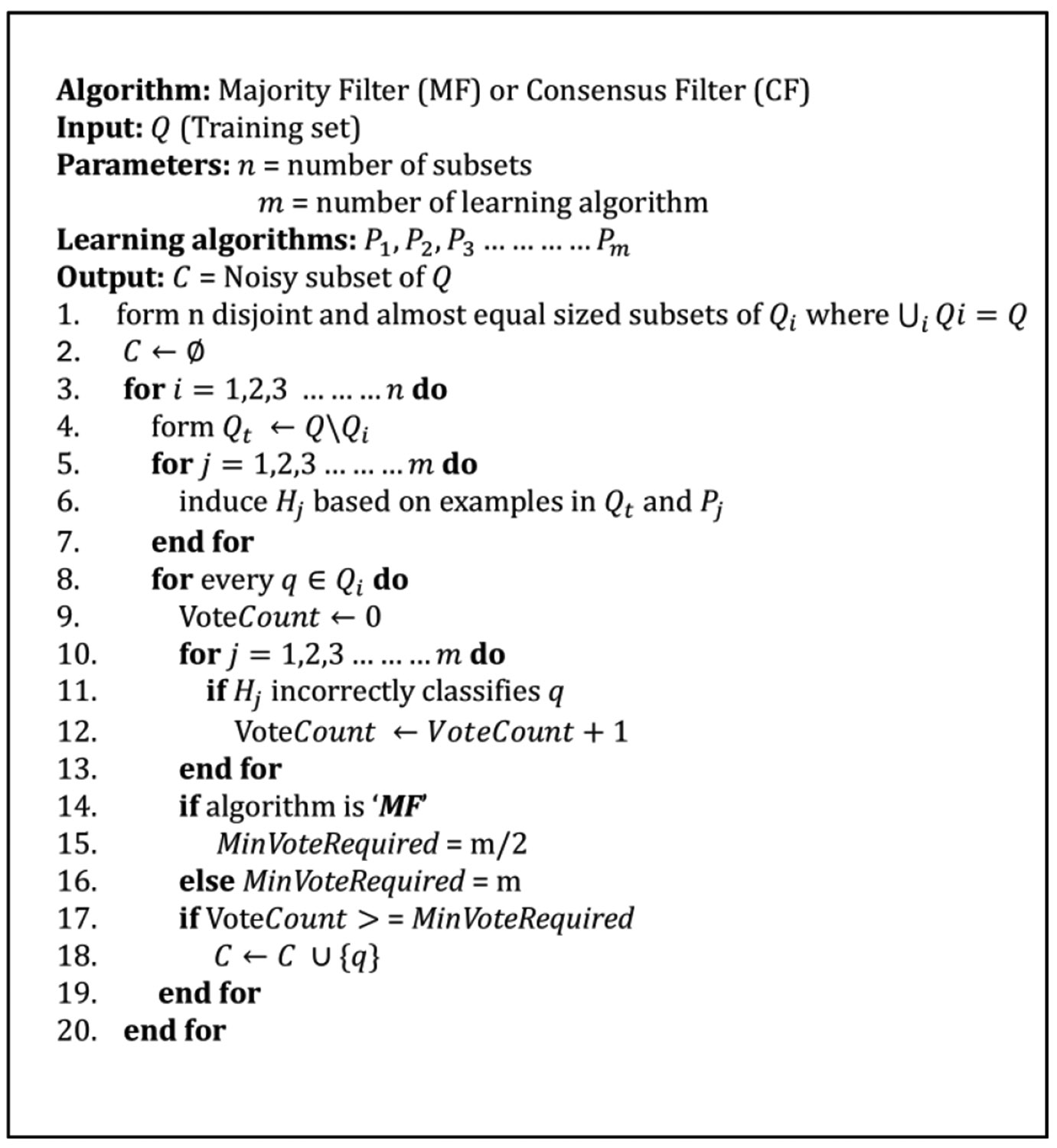
Algorithm for majority filtering (MF) and consensus filtering (CF) for m number of fine-tuned models. An instance is tagged as a mislabel if it passes the minimum vote required. For MF, the minimum vote required is m/2, whereas for CF it is m. C is the set of noisy instances/mislabels, where each mislabeled data, q is stored.

A summary of both filtering methods is presented in Fig. 4. The whole training dataset Q was divided into an equal number (n) of subsets. In our case, the total number of subsets was kept at n = 10. m number of fine-tuned learning algorithms $\left(P_{1},P_{2},P_{3},\ldots \ldots ,P_{m}\right)$ are used to form the ensemble. In each iteration, nine subsets were concatenated to form the training data, while the remaining subset was used for testing. Induced hypothesis (classifier), $H_{j}$ is found by training $P_{j}$ using training data $Q_{t}$. In the prediction part, each classifier $\left(FTM_{j}\right)$ predicted on the testing subset, and their votes were accumulated. If the vote count becomes more than half of the total number of classifiers, that particular instance was tagged as mislabeled data in MF and saved in the mislabeled set, C. So, for five classifier cases, the vote count should be more than or equal to three to be tagged as mislabeled in MF.

Unlike MF, in the case of CF, to tag an instance (q) as a mislabel, all the m classifiers should agree on that. The process of creating the subset and training remains the same as MF. This model was validated and run for different datasets in the next sections.

## IV. RESULTS AND DISCUSSION

### A. IDENTIFICATION OF MISLABELS IN THE MNIST DATASET

To evaluate the performance of our model, we applied it to the MNIST database [49], which consists of handwritten digit images (0-9). The dataset contains 60,000 training samples and 10,000 test samples, with each image a 28 × 28 grayscale matrix, yielding 784 features per image. For this study, we used only the training set for our model validation. Thus, the number of mislabels was counted for the training data set. For MNIST, we employed fine-tuned models developed based on ResNet-50, VGG-16, MobileNet V2, Inception V3, and ResNet-101. After applying our method to identify mislabeled instances within the MNIST dataset, we detected a significant number of mislabeled samples. We conducted experiments using voting criteria of 3/5 (three out of five), 4/5 (four out of five), and 5/5 (five out of five), with the corresponding results presented in Table 2. However, after manually reviewing the identified mislabeled instances, we found that not all cases were actual mislabels. Some images were ambiguous, while others had inherent quality issues, contributing to the detected mislabel count.

**FIGURE 5.**
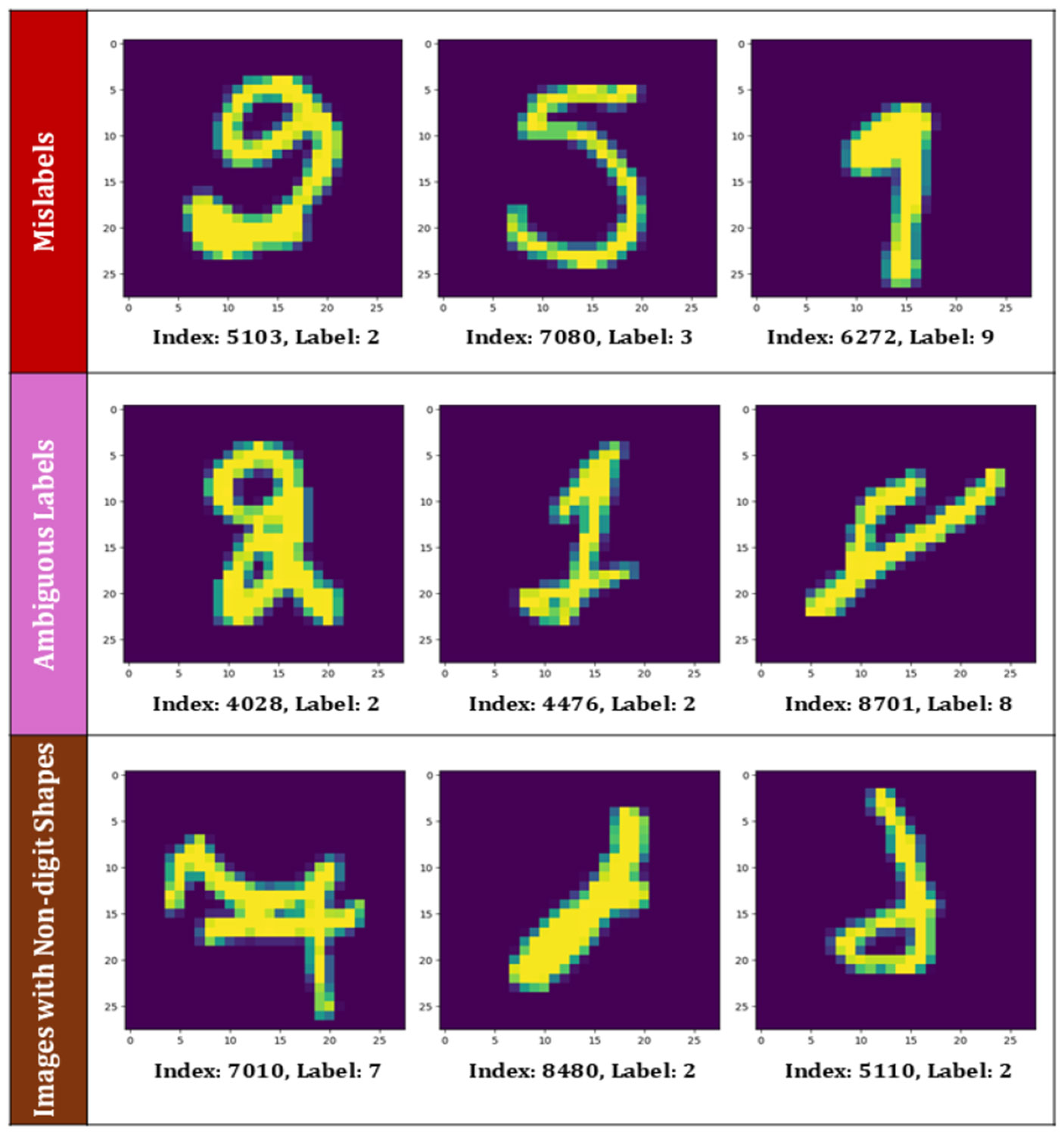
Different types of mislabels found in MNIST data. These include some clear mislabels, some are ambiguous, and some involve non-digit like shapes.

Figure 5 illustrates three types of mislabeled instances. The first row shows clear mislabels, along with their index positions and assigned labels. The second row presents ambiguous cases in which the correct label is uncertain or difficult to determine. The third row highlights images with distorted or non-digit-like shapes. Previous studies have attempted to quantify the number of mislabeled samples in the MNIST dataset. Zhang et al. [29] identified 675 mislabeled images in the MNIST dataset using ensemble-based filtering and manual inspection. In our validation with the fine-tuned ensemble, the proposed MF (4/5) approach detected 751 potential mislabels, which is reasonably close to the previously reported estimate.

The accurate identification of the number of mislabels in the MNIST dataset is difficult due to the inherent ambiguity of some digits (e.g., 4 vs 9, 3 vs 5), as shown in Fig. 5. In our study, all samples flagged as mislabels were manually reviewed. Based on the true labels of the flagged mislabeled images, the precision value was found to be in the range of 0.87~0.88 for MF (4/5 voting methods) and CF (5/5 voting methods), where the precision is defined as the ratio of true positives to total positives (both true and false positives). *This precision level is considered sufficient for a preprocessing quality-control framework, where the primary objective is to improve overall dataset integrity rather than achieve perfect separation of borderline cases. Furthermore, the similarity between the 4/5 and 5/5 cases suggests that the majority of identified mislabels are consistently detected across models, indicating robustness of the voting framework.* However, in high-stakes biomedical contexts such as AAV signal classification, it is preferable to conservatively flag a small number of ambiguous samples rather than retain potentially incorrect labels that could bias downstream model training.

To further evaluate the reliability of our method, we introduced 50 forced mislabels into the MNIST dataset, and the model was run for both MF (3/5) and CF (5/5) settings. We observed that the ensemble successfully detected all artificially injected mislabeled samples in both cases. *These results demonstrate that the proposed filtering strategy is highly effective in identifying labeling errors while also capturing the difficult, ambiguous cases that naturally occur in handwritten digit datasets.* Next, we apply the filtering algorithm to the AAV dataset, developed through in-house experimentation.

### B. APPLICATION OF THE FILTERING ALGORITHM IN THE AAV DATASET

Although viruses cause numerous diseases in all living organisms, not all viruses are harmful. In fact, appropriately designed viruses can be used for drug delivery and vaccine development. For instance, in recent years, adeno-associated viruses (AAV) have been used for gene delivery to treat hereditary blindness [50]. For safe and effective treatment, an appropriate amount of gene (either single-stranded or double-stranded) must be loaded in each virus particle [39], [51], [52]. Owing to the very small size (less than 20 nm), it is very difficult to characterize the content of an AAV. Lately, our group has developed a solid-state nanopore to characterize different types of AAVs [53], [54]. In this study, we developed an AAV dataset from the solid-state nanopore experiments to test out our filtering algorithm.

#### 1) EXPERIMENTAL PROTOCOLS

**FIGURE 6.**
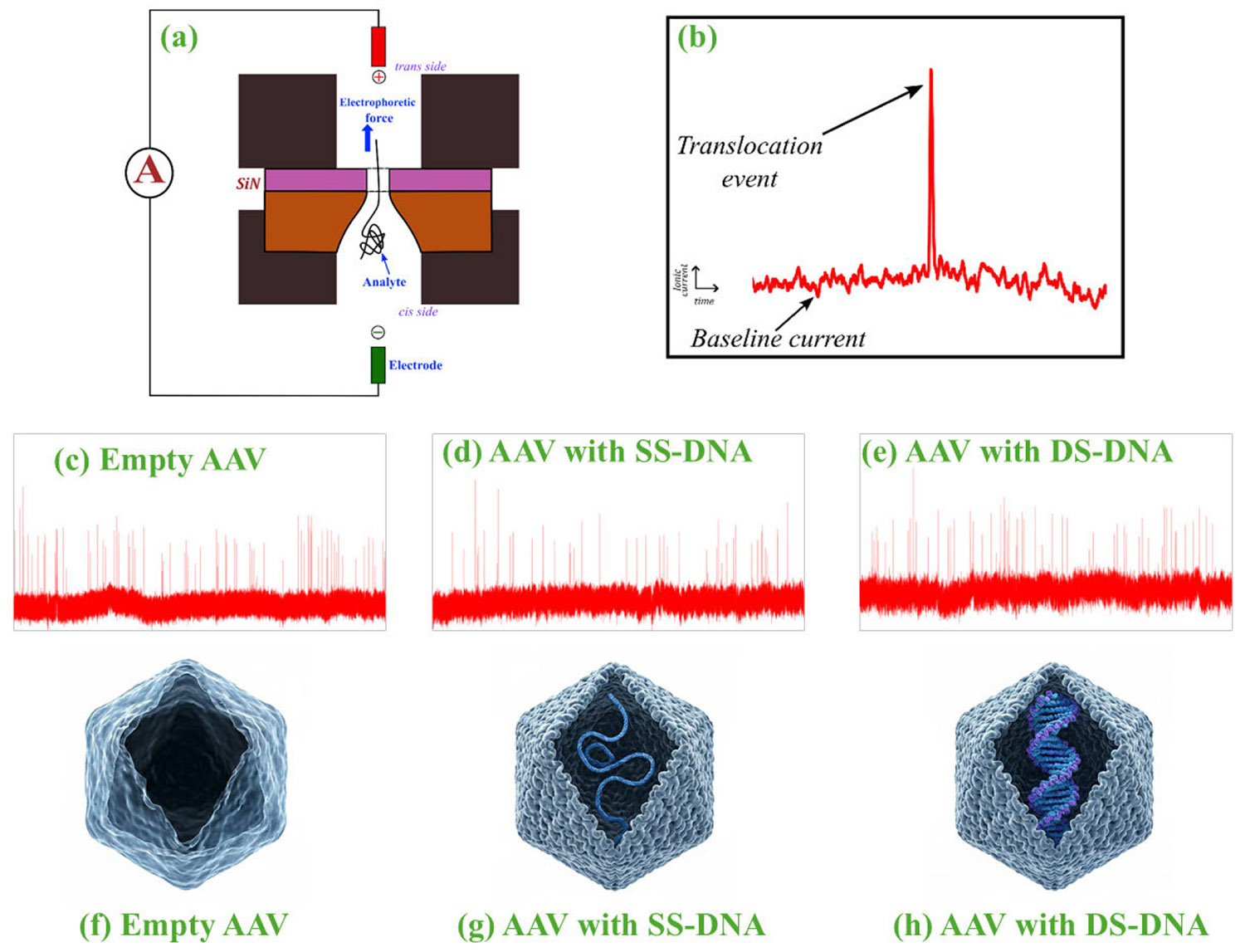
(a) A solid-state nanopore experiment setup consisting of a 12 nm thick silicon nitride chip. AAVs are placed on the cis side, and they get translocated to the trans side due to the effect of the electrokinetic forces. Ionic current changes are recorded for these translocation events (b). Representative images of ionic current distribution for (c) empty AAV, (d) AAV filled with single-stranded (SS) DNA, and (e ) AAV filled with double-stranded (DS) DNA. (f), (g), (h) present structural variations between the biologically distinctive AAV particle states. Empty capsids contain no genetic material, ssDNA particles comprise a single-stranded viral genome, and dsDNA particles hold a replicated double-stranded genome. These structural differences affect the ionic current signatures recorded during nanopore experiments, producing unique time-series patterns and corresponding image representations.

The concept of nanopore sequencing was first introduced in the 1980s, and since then, there has been huge development and refinement in this area [55]. These nanopores are typically fabricated on very thin (~50 nm) synthetic membranes, which create a separation layer between two chambers connected by electrolytes. Under the impact of an applied electric field, particles begin to translocate from one chamber to another, as shown in Figure 6(a). This translocation event through the nanohole creates changes in the ionic current signals. Usually, a rising or falling edge is observed on the base ionic current signal during a translocation event (Fig. 6(b)). We specifically recorded the time series data of ionic current for three types of AAV samples: empty AAV (Fig. 6(c)), AAV filled with single-stranded **(SS)** DNA (Fig. 6(d)), and AAV filled with double-stranded **(DS)** DNA (Fig. 6(e)), when they are translocated through the nanopore under electrokinetic force. Their physical structures are presented in Fig. 6(f), 6(g), and 6(h).

#### 2) AAV DATA PREPROCESSING

All the pre-trained CNN models described earlier (Section III-A) accept data as 3-channel images. Thus, some preprocessing of data is needed before inputting them into the pre-trained models for cross-validation training. In our solid-state nanopore experiments, raw current flow data are recorded in axon binary format (ABF) at a sampling frequency of 250 MHz. These time series data contain time and current signals as two separate series. To create an AAV database from a limited number of experiments, the entire time-series data are segmented, and plots are generated for each segment. The length of each segment was 1 sec. So, there are 250,000 data points that fall into one segment. Each segment of data is plotted in color images, which makes them 3-channel data with RGB values. Also, the image dimensions are made compatible with the requirements of the pre-trained models described in section III-A.

**FIGURE 7.**
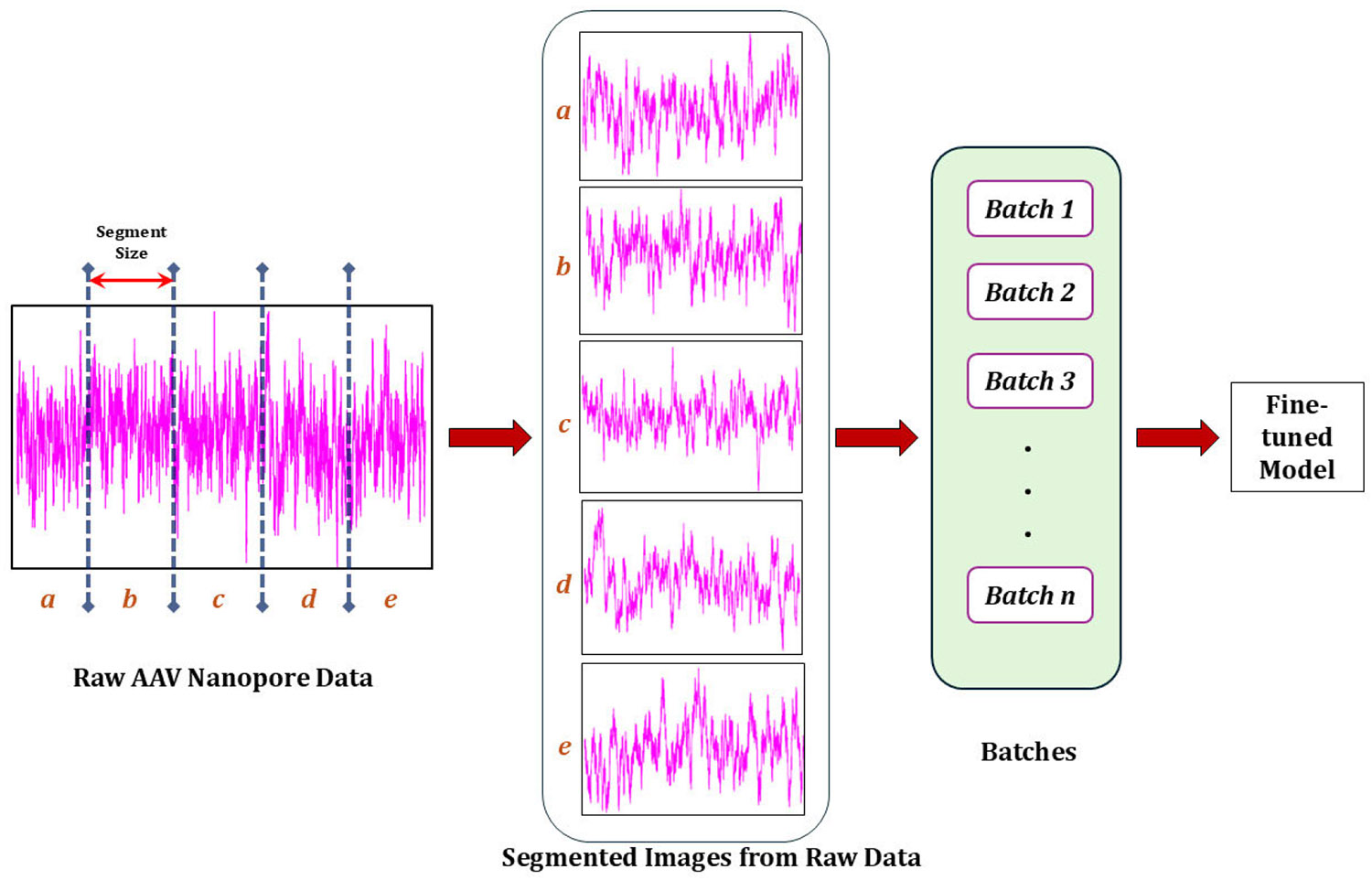
Data preprocessing involves segmentation of the raw data. Segmented plots and their labels are fed as batches into the machine learning model for training. Here, 1-second segments are used to create images. The nanopore experiment was done at 100 mV for three types of AAVs: single-stranded (SS), double-stranded (DS), and empty DNA.

Figure 7 presents the summary of the preprocessing. Here, a small section of the raw data is shown for visualizing the process. Small segments of equal size are made from this data to form images for the AAV dataset, and multiple images are used to form a batch. Usually, a batch size of 32 is used for machine learning training. A summary of the 2D images for the AAV data is given in Table 3. Training and testing dataset sizes are also shown and used to compute accuracy for individual classifiers in later sections of the paper. Due to the sensitive applications of these viruses in gene delivery, separate careful experiments are performed for each type of analyte: Empty, SS-DNA, and DS-DNA. *Owing to the carefully crafted experiments, these AAV nanopore data are considered highly pure without any mislabeled instances. To evaluate our filtering model’s effectiveness, we introduced intentional (random) mislabels into an otherwise pure AAV dataset. Our goal was to determine whether our model could accurately detect all these mislabeled instances.*

To thoroughly evaluate our model’s ability to detect mislabeled instances, we examined the effects of voting methods and Softmax confidence thresholds. Five different voting strategies were employed: 3/5, 4/5, 5/5, 2/3, and 3/3, each determining the minimum agreement required to classify a sample as a mislabel. Additionally, we adjusted the Softmax confidence threshold across multiple values to analyze its impact on model performance.

#### 3) PERFORMANCE EVALUATION FOR AAV DATA

Before evaluating the transfer learning models, we also tested classical clustering techniques such as KNN and k-means for mislabel detection. *Compared with the pre-trained models, both clustering methods achieved low accuracy (KNN: 70.52%; K-means: 49.86%) on the AAV dataset; representative results are provided in* Appendix A. Due to the resulting low accuracy, they will perform poorly at mislabel identification and true-label prediction. *These limitations motivated a shift toward pretrained deep models, whose performance is examined next.*

**FIGURE 8.**
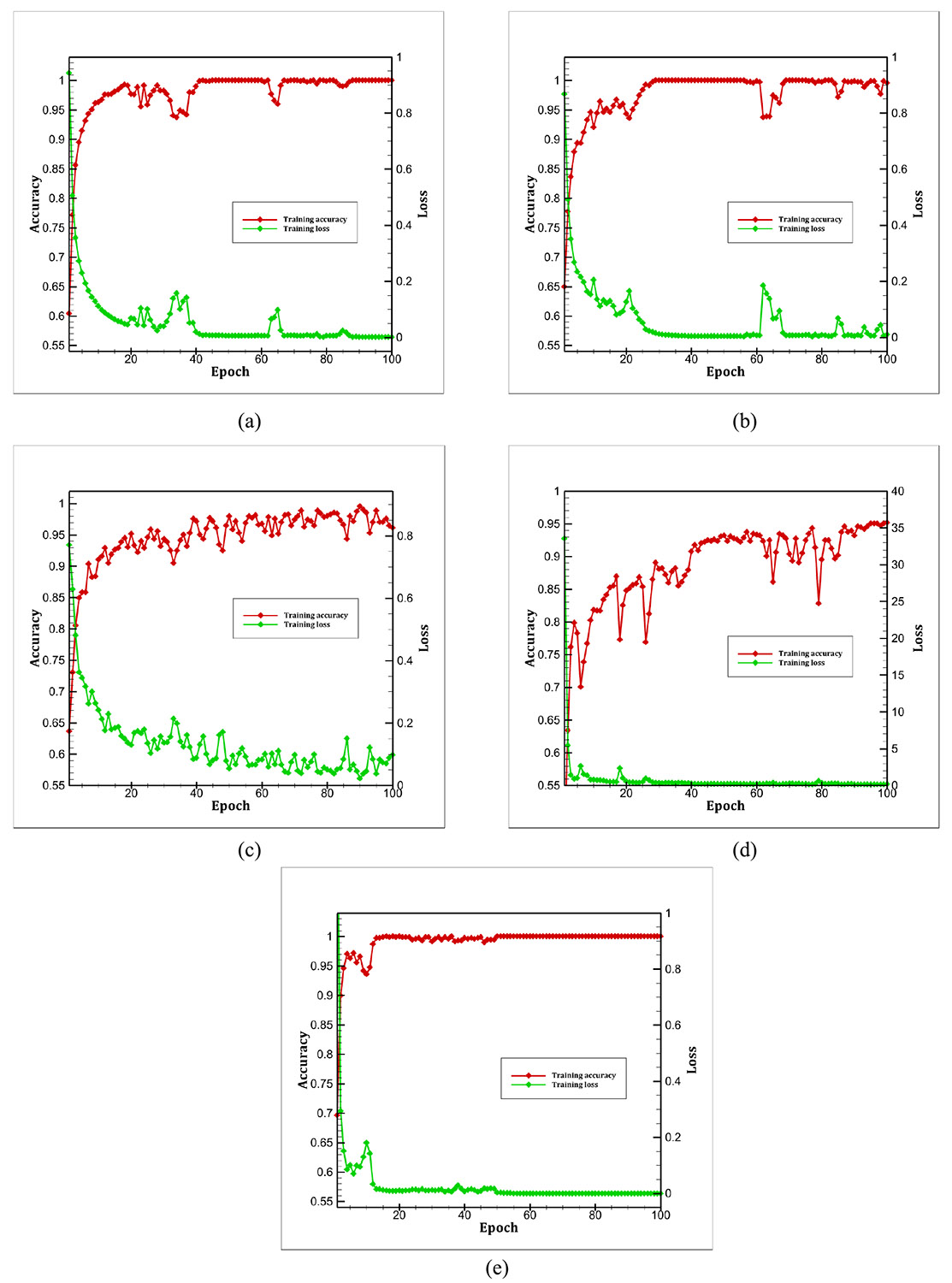
The individual training curves for all five classifiers: (a) ResNet-50, (b) ResNet-101, (c) EfficientNet B0, (d) Inception V3, and (e) VGG-16. Training accuracy and training loss are presented as metrics here. Results are presented for 100 epochs in each case.

The individual training curves for all classifiers trained on the AAV dataset are presented in Fig. 8. A consistent trend of decreasing loss and increasing accuracy is observed across all models, indicating effective learning. However, the shapes of the curves vary depending on the underlying architecture of each pre-trained model. Both ResNet-50 and ResNet-101 produced similar training curves, which is expected given their shared use of residual blocks and architectural design. The inception model exhibited the highest initial loss but rapidly converged to a low value within just a few epochs. Similarly, VGG-16 demonstrated fast convergence, with the loss and accuracy curves flattening out after approximately 20 epochs.

Next, we evaluate the algorithm’s effectiveness by introducing 20 mislabels at random into the dataset, following the scheme presented in Figure 1. *Here, instead of strictly following Eq. (2) for prediction, we introduced a confidence threshold to refine the voting mechanism. A classifier’s vote was considered for a particular image only if it misclassified that image with a minimum confidence level, meaning that the Softmax probability of the incorrect class exceeded the set threshold.* For the AAV dataset, 760 images were divided into 10 subsets, each having 76 images. In each round, the test set is decided based on the round number, while the remaining data go to the training set. After training is finished in a round, the trained classifiers (*FTM* : 1 *to m*) predict on the test data and give a probability distribution of the three classes. *As mentioned earlier, a prediction can’t participate in the voting section if the maximum value of the probability distribution vector is not greater than the confidence threshold value* (*δ*). *This approach ensures that only high-confidence predictions contribute to the voting process, reducing the influence of uncertain predictions and improving the reliability of mislabeled sample detection.* Confidence thresholds of 0.5, 0.6, 0.7, 0.8, and 0.9 were used here for this study.

The numbers of false positives and false negatives for the run are listed in Table 4 across different voting methods and confidence thresholds. *In the context of AAV nanopore data, false positives and false negatives directly impact both safety and therapeutic effectiveness. False positives allow empty or contaminated capsids to be misclassified as valid AAVs, risking patient safety, while false negatives discard functional AAV vectors, reducing treatment efficacy.*

The performance of the ensemble model was evaluated using precision, recall, and F1 score parameters, which are calculated from the true positives (TP), false positives (FP), and false negatives (FN) as:

(4) $$\text{Precision}=\frac{\text{TP}}{\text{TP}+\text{FP}}$$

(5) $$\text{Recall}=\frac{\text{TP}}{\text{TP}+\text{FN}}$$

(6) $$F1\;\text{score}=2*\frac{\text{Precision}*\text{Recall}}{\text{Precision}+\text{Recall}}$$

**FIGURE 9.**
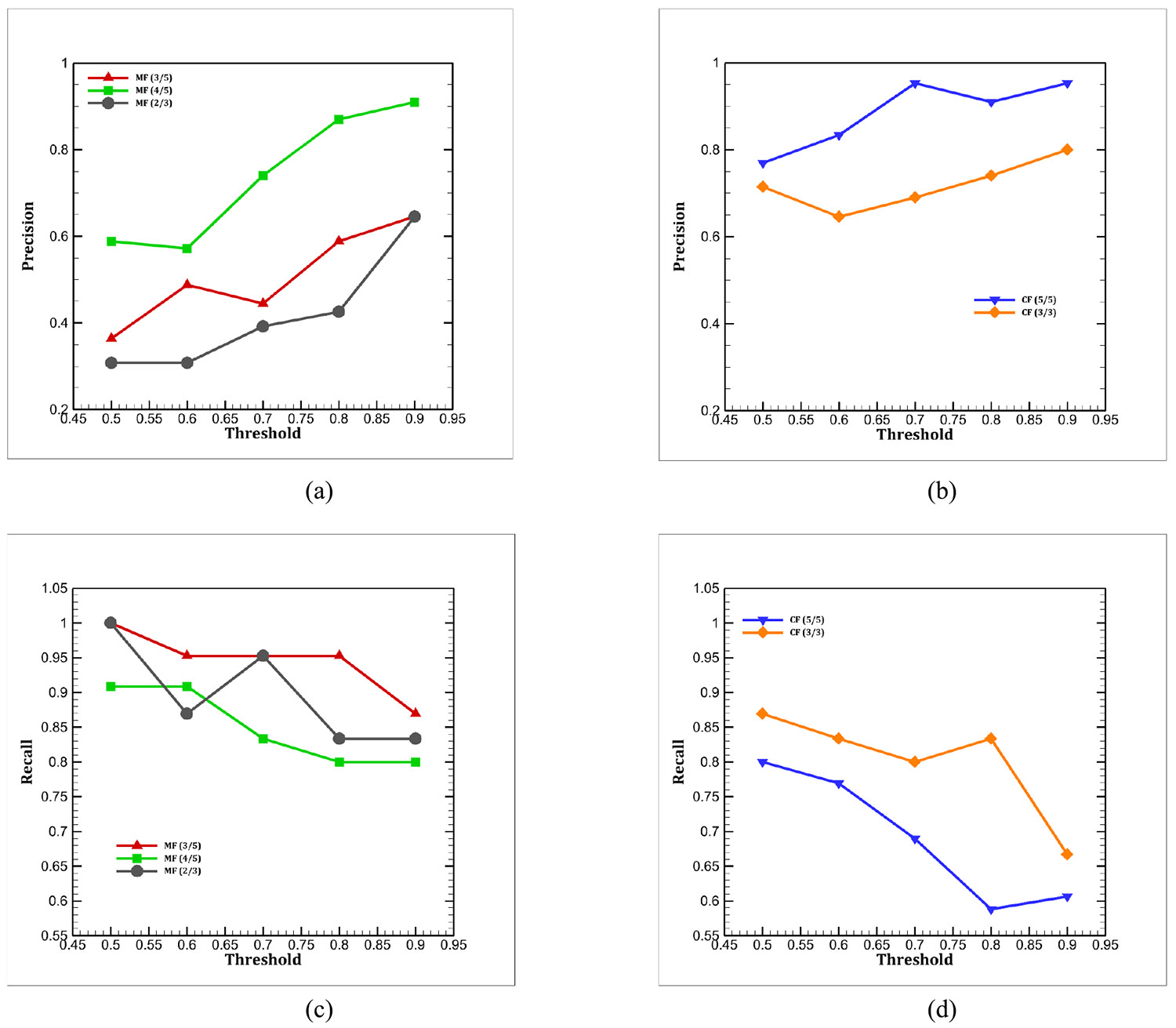
Precision and recall values for five different voting methods with the change of confidence thresholds: (a) Precision for majority filtering, (b) precision for consensus filtering, (c) recall for majority filtering, and (d) recall for consensus filtering.

Precision reflects how well the model avoids FPs, recall measures its ability to minimize FNs, and the F1 score balances the two. *Since both errors (precision and recall) are costly in biomedical applications, especially gene therapy, evaluating models with these metrics is essential to ensure reliable and safe classification.* Figure 9 presents the precision and recall distributions for the different confidence thresholds investigated here.

The ensemble model shows an overall increasing trend in precision as the confidence threshold rises. The consensus filtering (CF) methods achieved higher precision scores, with the highest value obtained by CF (5/5) at a 0.9 confidence threshold. Conversely, recall exhibits a decreasing trend with increasing confidence. In this metric, the majority filtering (MF) method performed better, with the highest recall achieved by MF (3/5). It is noteworthy that although both CF (5/5) and CF (3/3) yield high precision, they also result in the lowest recall. *This suggests that CF is preferable when the minimization of false positives is critical, whereas MF is more effective in reducing false negatives*.

**FIGURE 10.**
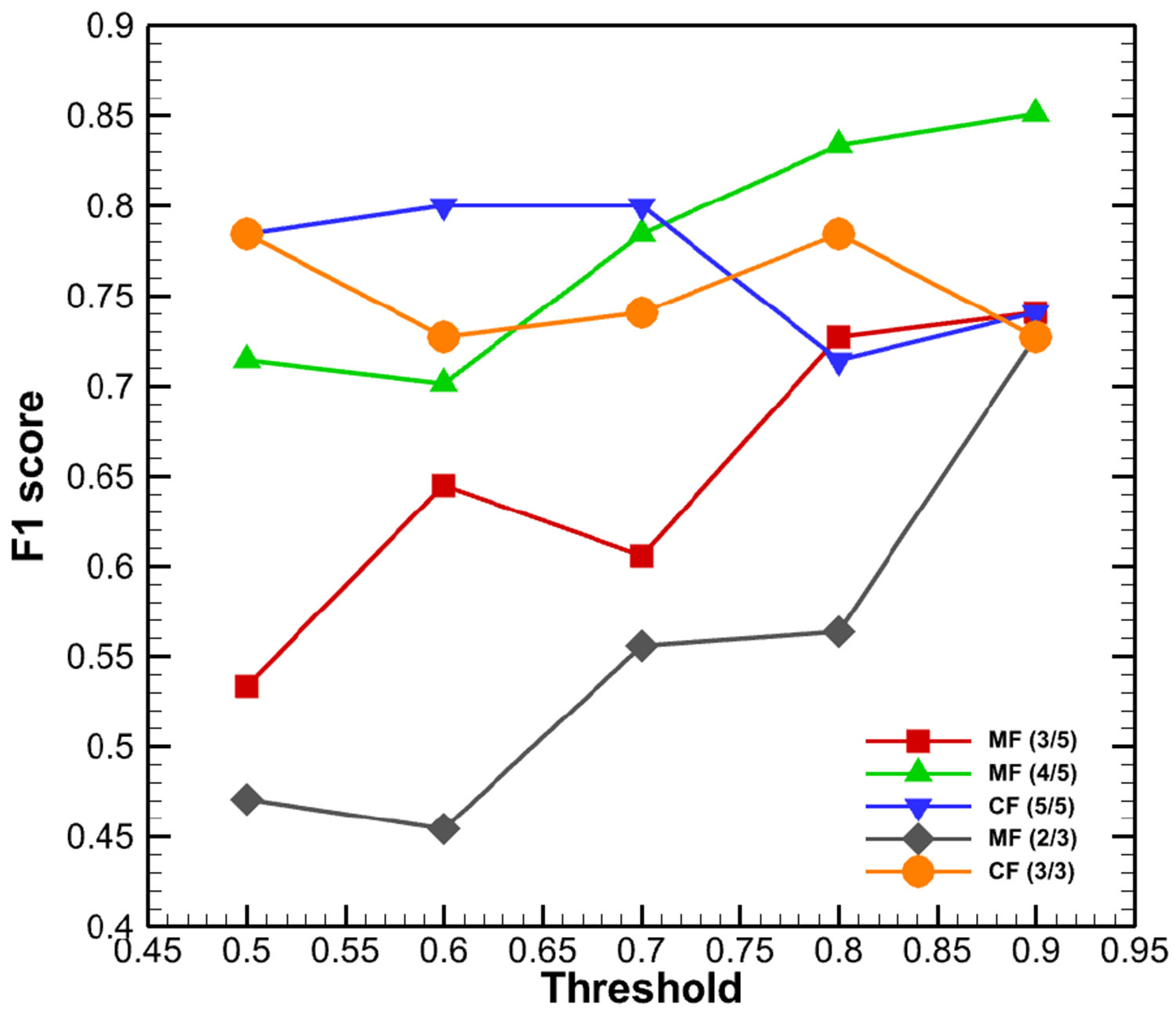
F1 score for five different voting methods with the change of confidence thresholds.

To evaluate the combined effect of precision and recall, the F1 score was used. Figure 10 presents the F1 scores for all five voting strategies. Among them, MF (4/5) achieved the highest F1 score at the 0.9 confidence threshold, indicating a balanced performance with relatively low false positives and false negatives under this configuration.

#### 4) IN-DEPTH PREDICTION ANALYSIS

To evaluate the performance of individual classifiers, predictions from each model were recorded during a run. Table 5 presents the results for a setup with five classifiers, using a 4/5 majority voting scheme at a threshold of 0.80. It can be observed that although the data labels in this experiment were intentionally modified, the classifiers generally recovered the true labels in most cases. Occasionally, one or two classifiers failed to predict the original label correctly; however, this did not prevent the ensemble model from successfully identifying these samples as mislabels. A comparable scenario is shown in Table 6 for the 2/3 voting scheme with a threshold of 0.50.

Similar to the previous case, most mislabels were correctly detected, with errors arising primarily from the Inception classifier. To further investigate, Inception was replaced with MobileNet, and results for both the 4/5 and 2/3 voting schemes are reported in Tables 7 and 8, respectively, for comparison, using the same threshold values as before.

In both cases, the number of incorrect predictions made by individual classifiers was reduced. Nevertheless, a few mislabeled samples remained undetected. Upon examining the indices of these samples, it was observed that the two unidentified mislabels in the 2/3 majority case were also missed in the 4/5 majority case. However, due to the extremely subtle and visually ambiguous nature of the signals (Ref to Fig. 6), a detailed manual analysis of these missed signals is challenging. Still, a confidence score analysis was performed for all of the injected mislabels. Across the 20 injected mislabels, the average confidence score for detected mislabels was 0.9607. On the other hand, the confidence scores for two missed mislabels were 0.8549 and 0.7628. These values are significantly lower than the overall average confidence of the detected mislabels, indicating that the missed samples were associated with comparatively weaker model certainty. This suggests two key observations:

- **Reduced separability in feature space** – The missed samples likely lie closer to class boundaries, where signal morphology overlaps between categories.
- **Lower ensemble agreement strength** – While these samples were mislabeled, their internal feature representation remained sufficiently consistent with their assigned label to avoid strong consensus-based rejection.

In other words, the missed mislabels were not arbitrary failures but correspond to borderline cases with lower discriminative confidence.

It is important to note that the quantitative assessment was possible due to the injection of artificial mislabels in the highly curated AAV data. Since the complexity and structure of the real world label noise could be different, the reported results should be interpreted within this context.

#### 5) PREDICTION ACCURACY AND RUNTIME ANALYSIS

**FIGURE 11.**
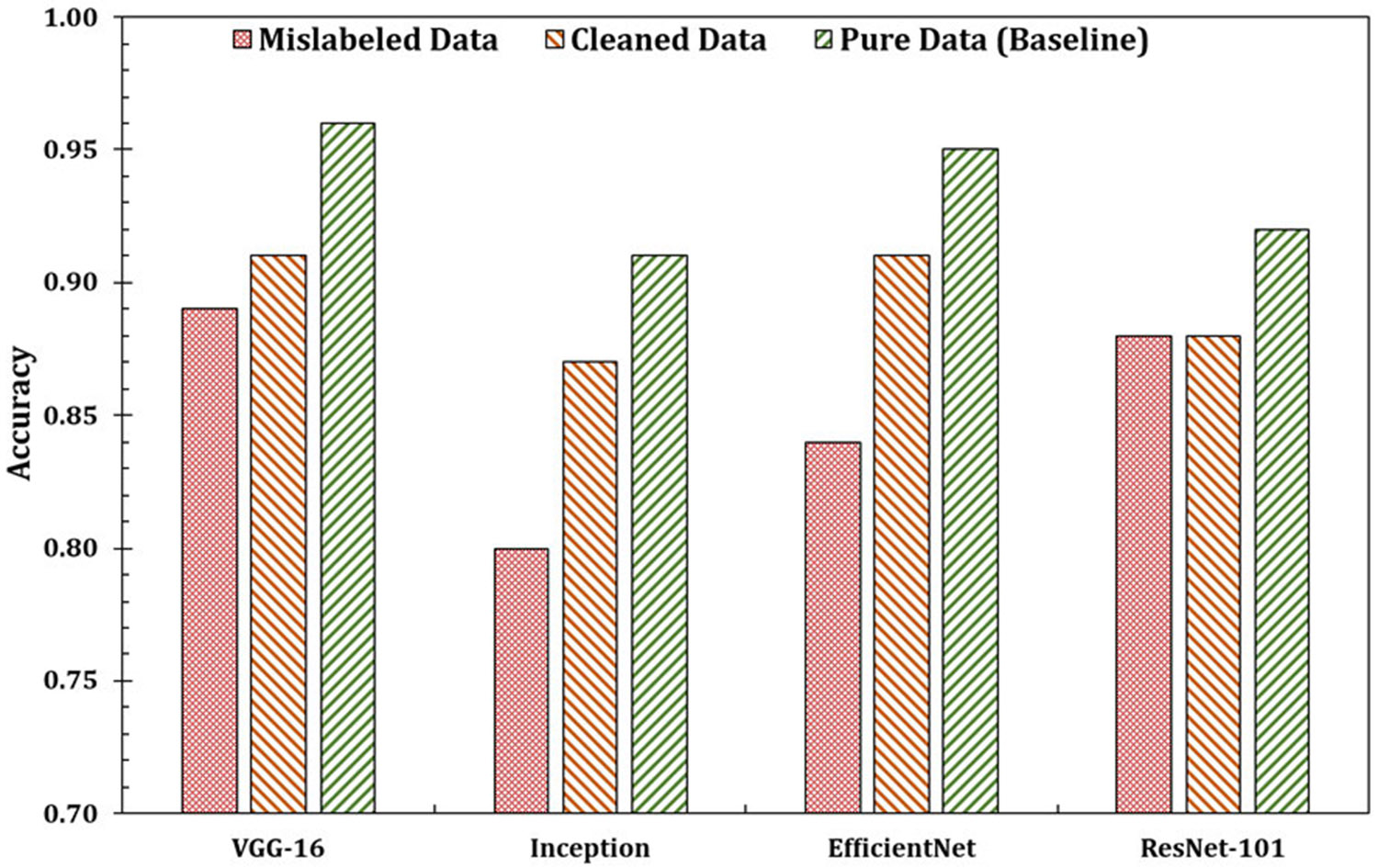
Comparison of validation accuracy between mislabeled data, cleaned data, and pure data. Results are presented for five pre-trained models, with the pure data serving as the baseline comparison.

Next, we compare the performance of each classifier for clean (pure) data and the data containing (randomly introduced) mislabels (Fig. 11). As expected, we obtained better model accuracy for the clean/pure data for each classifier. The results presented in Fig. 11 show the validation accuracy of the base learner models. The highest validation accuracy was achieved using the pure dataset, while the addition of mislabeled samples led to a noticeable decline in performance. When the mislabeled data were identified and corrected using the ensemble model, accuracy improved, demonstrating the effectiveness of the proposed approach in cleaning noisy data. However, this trend did not hold for ResNet-101. Although the ResNet-101 model performed better on the pure dataset compared to the mislabeled one, no significant improvement in accuracy was observed after cleaning. This behavior suggests that the deeper residual architecture may already be relatively resilient to small amounts of label noise, leading to minimal observable gains after filtering.

**FIGURE 12.**
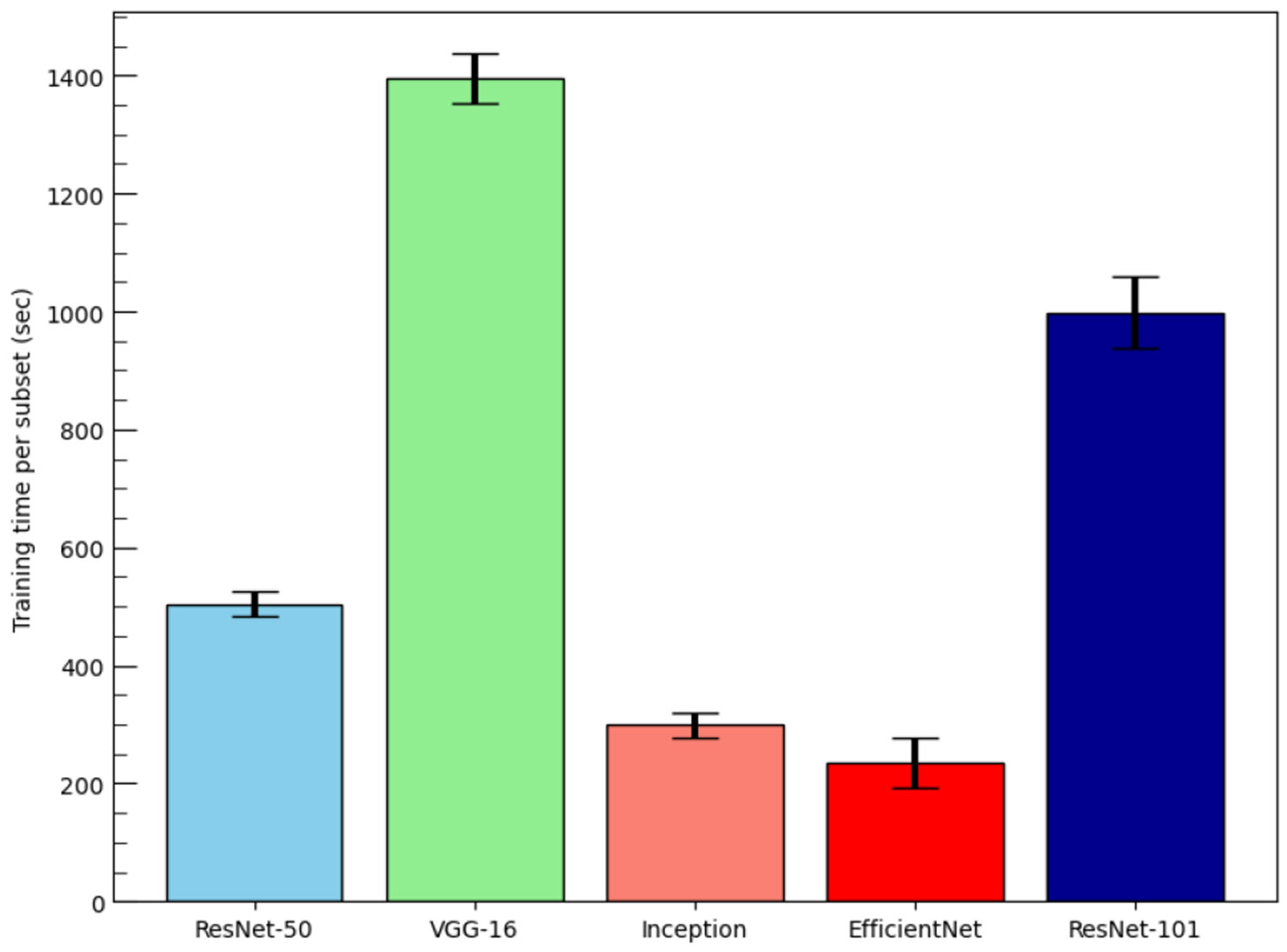
Comparison of training time per subset for each of the five classifiers. The error bars indicate the range of results obtained from five replicas.

In any classification task, computing time is as important as model accuracy in selecting a classifier. Figure 12 illustrates the training time per subset (mentioned in Fig. 1) for each classifier. Here, all runs were performed on a computer with an Intel Core i7–2.1 GHz CPU and 64 GB of RAM. All models were trained for 30 epochs using a fixed learning rate of 0.001. Due to its large number of trainable parameters (as shown in Table 1), VGG-16 required the longest training time among all classifiers. In contrast, EfficientNet completed training in the shortest time, reflecting its lightweight architecture. Additionally, a noticeable increase in training time is observed when moving from ResNet-50 to ResNet-101, which can be attributed to the significantly greater depth of ResNet-101.

The total runtime breakdown is presented in Table 9 for the five-classifier and three-classifier ensemble models. It is evident that VGG-16 consumed the majority of the overall computation time. Specifically, it accounted for 40.22% of the total runtime in the five-classifier ensemble and 62.91% in the three-classifier configuration. Additionally, in both cases, nearly 99% of the total runtime was spent on training, with only a small portion allocated to testing and post-processing. *These results provide insight into identifying combinations of classifiers and configurations that are not only runtime-efficient but also effective at detecting mislabeled samples, with low false-positive and false-negative rates. Moreover, the computational costs mostly refer to the offline cleaning stage and don’t affect the other downstream tasks.*

#### 6) MISLABEL DETECTION FOR BALANCED DATA

The AAV dataset presented in Table 3 is imbalanced, with the “Single” class containing significantly more samples than the other two classes. Such an imbalance can bias the model’s learning process, leading to inflated performance for majority classes while underrepresenting minority ones. To more reliably assess model performance, it is necessary to evaluate results on a balanced dataset in which each of the three classes contains an equal number of samples. This can be achieved by selecting the same time range from each class; however, this approach limits the dataset size to that of the shortest available time series.

A balanced dataset was constructed by extracting a 120-second segment from each of the three classes, as this is the maximum overlapping interval among the classes in this dataset. The corresponding results are presented in Table 10. Our in-depth analysis indicates that all 20 mislabels were successfully identified by the model, whereas some had been missed in earlier runs on the unbalanced dataset.

A summary of the results for both the original unbalanced dataset and the newly created balanced dataset is provided in Table 11. The number of incorrect predictions made by individual classifiers increased on the balanced dataset, likely due to the reduced overall dataset size. However, a higher count of individual mispredictions does not directly translate to errors in the final ensemble decision. In many cases, the correct predictions from the majority of classifiers outweighed these individual errors, allowing the ensemble to produce an accurate final label.

#### 7) ENSEMBLE DESIGN CONSIDERATIONS FOR PRACTICAL DEPLOYMENT

To evaluate a more computationally efficient alternative, we also tested a near-homogeneous ensemble (Appendix B) using multiple variants of a custom CNN model. *A fully homogeneous ensemble was not pursued, as it would closely resemble standard k-fold training and offer limited architectural diversity. This near-homogeneous configuration serves both as an efficiency comparison against the heterogeneous transfer-learning-based ensemble and as a simple deep learning baseline without pretrained features*. The detailed results for the near-homogeneous (simple CNN) experiment are provided in Appendix B. While the three-classifier configuration in the near-homogeneous setup is trained in less than 65 minutes, its mislabel-detection performance was consistently lower with a very high number of false positives (more than 20% of the sample size), suggesting the need for a heterogeneous ensemble.

This comparison highlights a clear efficiency–accuracy trade-off: the near-homogeneous ensemble may be suitable for resource-constrained settings or rapid preliminary data screening, whereas the heterogeneous ensemble is preferable when maximizing label reliability is critical, particularly for subtle or high-stakes biomedical datasets.

In addition to classical baselines, we also conducted an experiment using DivideMix [16] to evaluate its suitability for the present low-noise setting. A flow diagram of the DivideMix framework is presented in Appendix C. With 20 injected mislabels, DivideMix partitioned the dataset into 623 labeled and 137 unlabeled (mislabeled) samples, which is substantially higher than the number of injected noisy labels. This indicates that even under low-noise conditions, the method identifies a relatively large subset of samples as uncertain, reflecting the weakness of mixture-based noise modeling in small- to moderate-sized datasets. While DivideMix has demonstrated strong performance on large-scale, high-noise benchmarks, its noise estimation strategy relies on sufficient data volume and noise prevalence to reliably distinguish clean from noisy samples. *In contrast, biomedical datasets such as AAV nanopore data often involve small sample sizes, subtle inter-class differences, and low signal-to-noise ratios, making conservative, consensus-based majority filtering a more controlled and practical alternative for high-integrity applications.*

## V. CONCLUSION

With the increasing reliance on machine learning for critical classification tasks, maintaining dataset integrity is essential to ensure accurate and trustworthy predictions. Mislabels in training data can degrade model performance and introduce risks in sensitive applications such as medical diagnostics and viral classification. To address this challenge, we developed an ensemble-based mislabel detection method that enhances the reliability of downstream analyses.

The proposed approach integrates multiple pretrained CNN models with majority and consensus filtering strategies, leveraging architectural diversity to identify mislabeled samples. Validation on the MNIST dataset showed that the method could accurately detect mislabeled examples, including recovering all artificially injected mislabels. When applied to the AAV nanopore dataset—where artificial mislabels were introduced due to the dataset’s inherent purity—the ensemble successfully detected most mislabeled samples and predicted their correct labels. Experiments with balanced and unbalanced versions of the AAV dataset further revealed that a balanced dataset enabled complete mislabel recovery, while the majority filtering (4/5) voting scheme consistently outperformed simple majority and full consensus voting.

The proposed framework can be applied to real-world data pipelines. Specifically, the filtering mechanism can serve as an offline data-quality-assurance stage prior to final model deployment. While the use of multiple ensemble models increases computational cost during training, this process is inherently parallelizable across GPUs or distributed systems, enabling scalability for larger datasets. More importantly, once mislabels are identified and the dataset is refined, the filtering framework does not introduce additional inference latency in production environments. Therefore, the proposed method can be deployed as a preprocessing module that enhances label integrity without impacting operational runtime performance, making it suitable for real-world biomedical applications such as AAV signal classification. While comparisons were also made against some state-of-the-art noisy label methods (e.g. DivideMix), this work does not aim to establish comprehensive superiority over all existing noisy-label learning methods. Instead, the proposed method is designed as a complementary framework that is effective for a small dataset like AAV.

Despite its effectiveness, the method has several limitations. The use of multiple pretrained CNNs increases computational cost during cross-validation, which may raise scalability concerns for very large datasets without adequate computational resources. However, this overhead is incurred primarily during the offline data-cleaning stage and does not affect deployment-time inference latency. The selection of appropriate confidence thresholds may require dataset-specific tuning, and determining true mislabels in real-world datasets remains challenging due to ambiguous samples. Furthermore, while transfer learning enhances robustness, performance may vary for data distributions that differ substantially from ImageNet-trained feature representations, reflecting a trade-off between computational efficiency and detection reliability.

Overall, the results demonstrate that the proposed ensemble-filtering method is a simple yet powerful tool for improving dataset quality in domains where accurate labeling is critical. Future work will explore scalability to larger datasets, reduce computational overhead, and integrate advanced deep learning techniques for further refinement.

## Figures and Tables

**FIGURE 13.**
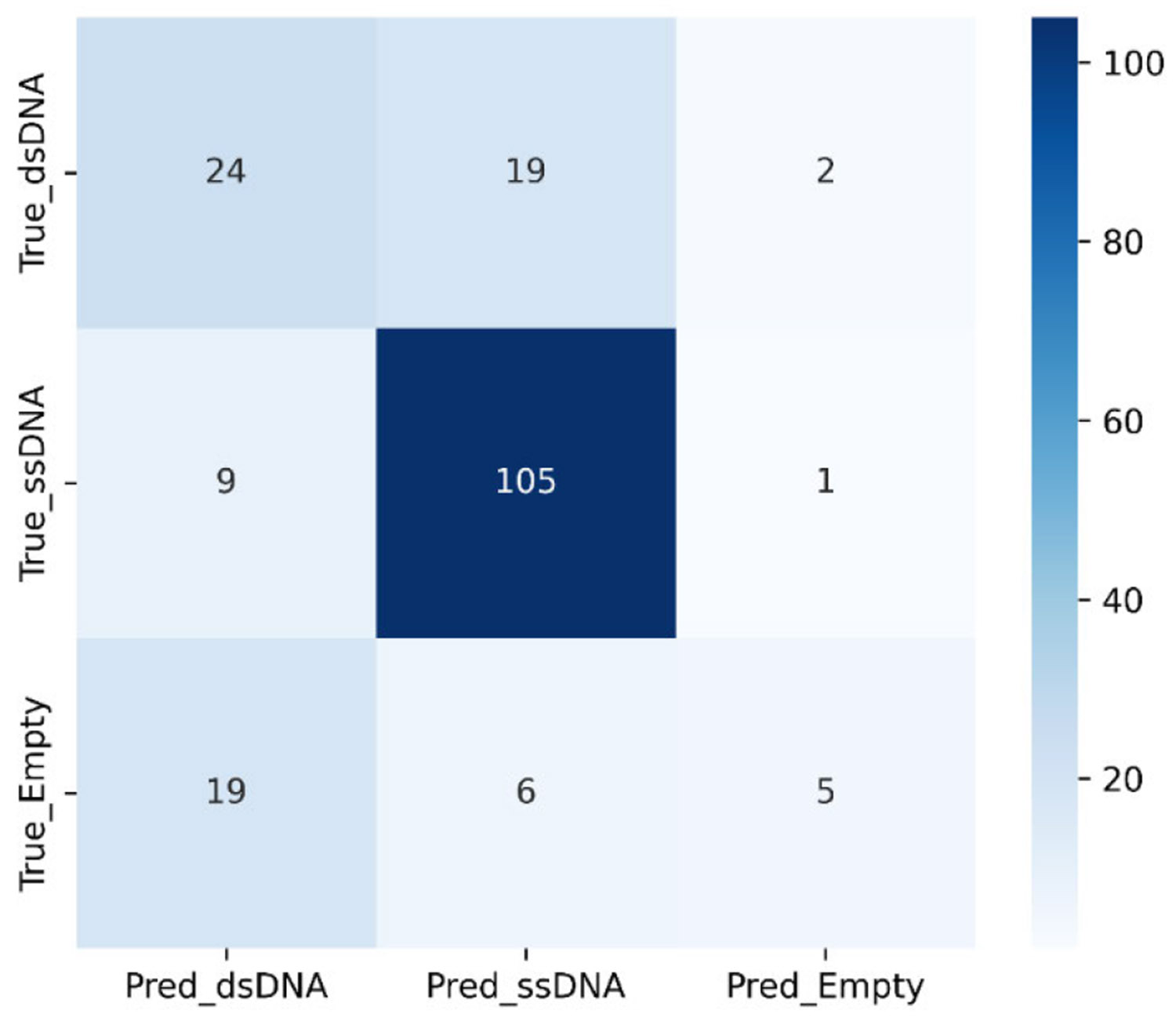
Confusion matrix for KNN with k = 5. The total AAV datasetsize was 760, and the test set was 25% of that.

**FIGURE 14.**
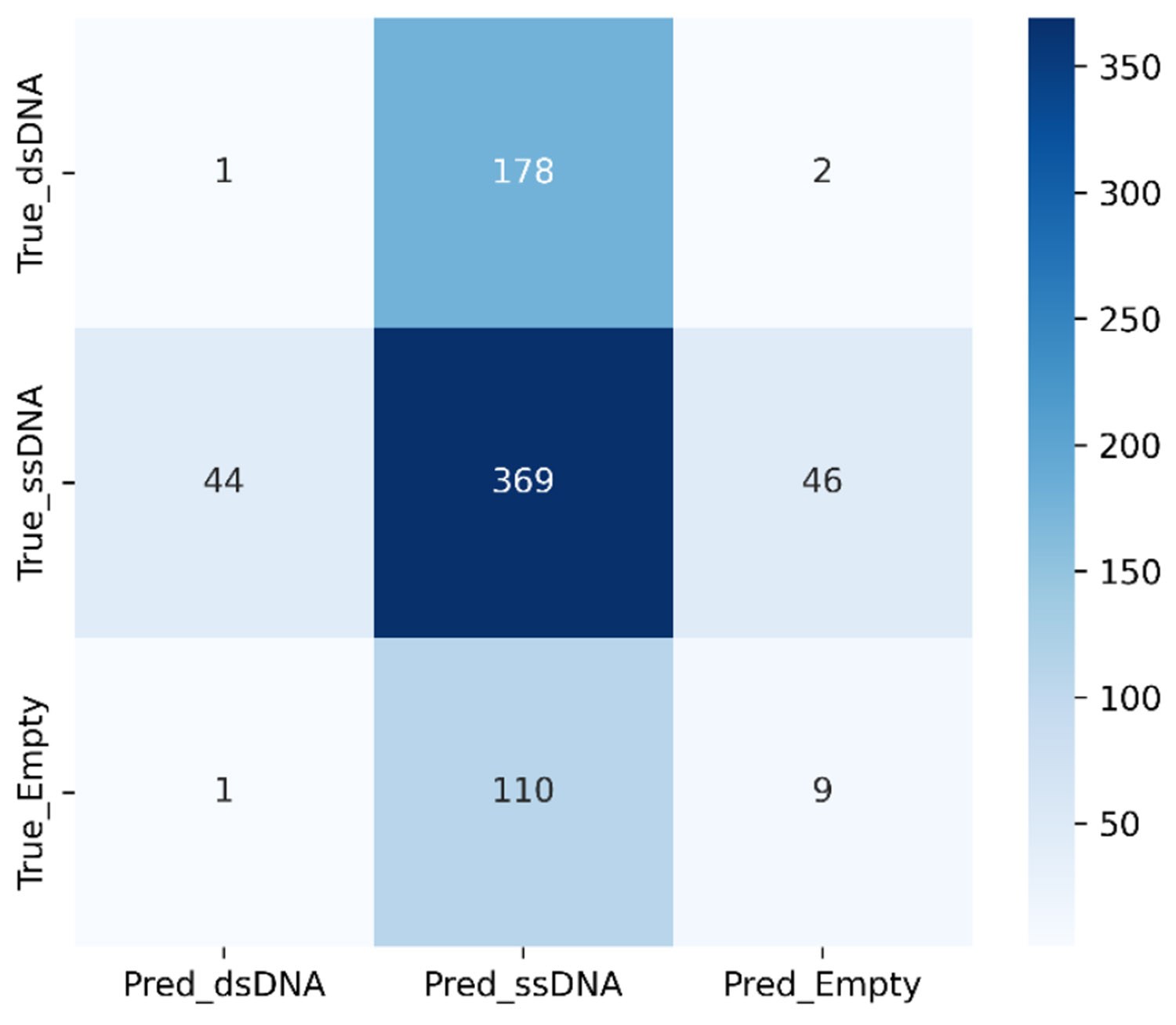
Confusion matrix for K-means for the total AAVdataset.

**FIGURE 15.**
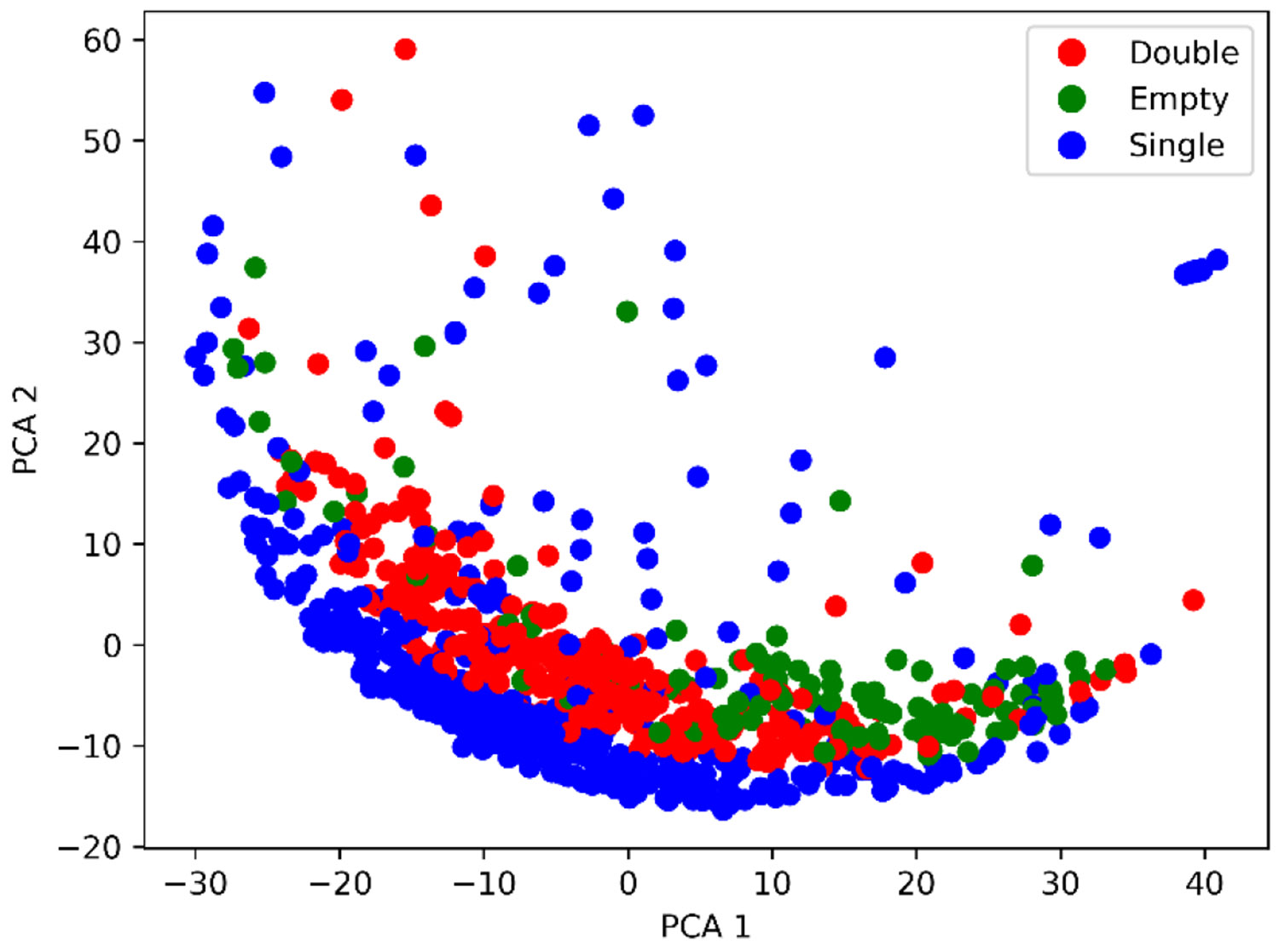
AAV data visualization using principal component analysis(PCA). PCA 1 and 2 indicate the two principal components.

**FIGURE 16.**
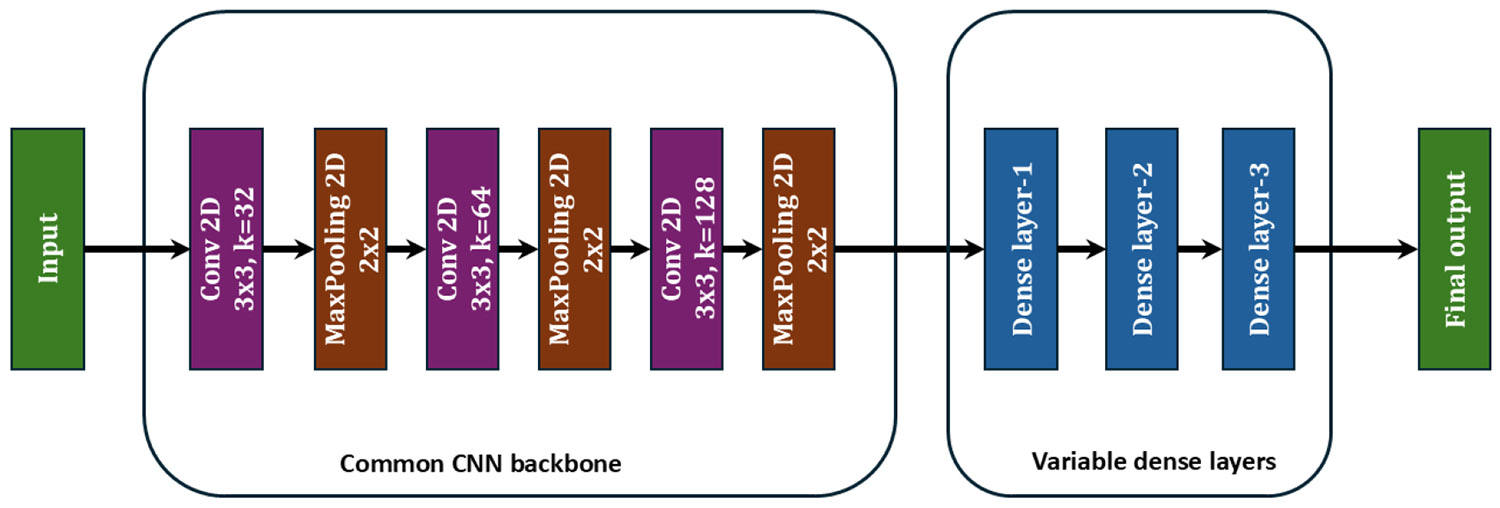
Customized CNN architectures used for homogeneous ensemblerun. The CNN backbone was kept the same, whereas the number of neurons in the dense layer was varied across the three models in the ensemble.

**FIGURE 17.**
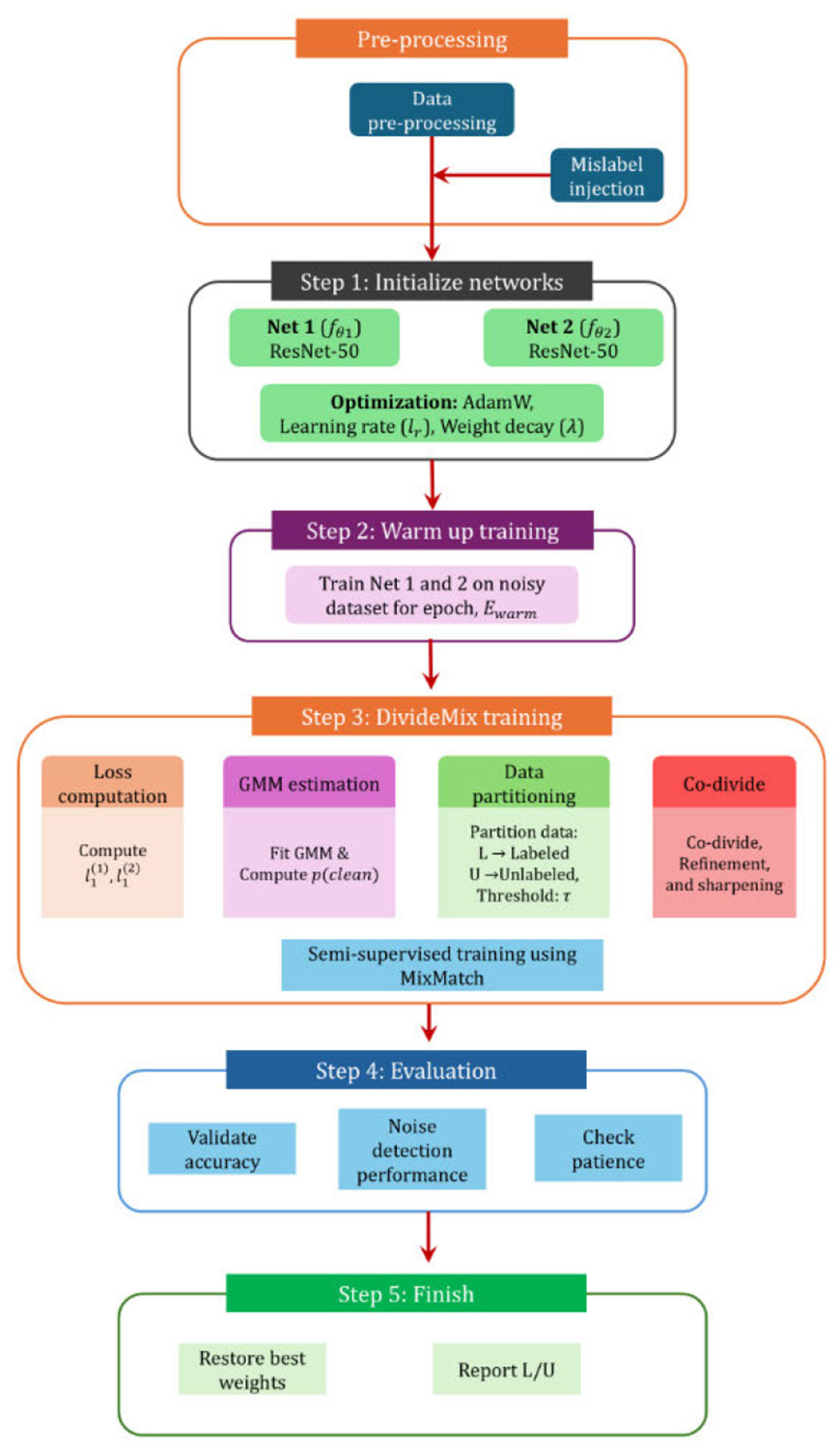
Flow of work for DivideMix implementation in the AAV dataset.

**TABLE 1.** Key characteristics of the pretrained models used in this work. These models were selected for their distinct architectural properties, which enhance the ensemble’s diversity.

Model	Number of layers	Approx, number of trainable parameters (millions)	Unique characteristics	Accuracy on ImageNet (top-1 accuracy)
ResNet-50	50	25.6	Residual blocks	76.0%
VGG-16	16	138	Small convolution filters	71.3%
Inception V3	48	23.9	Inception modules	78.1%
EfficientNet BO	18 (blocks/stages)	5.3	Compound coefficient scaling	76.3%
ResNet-101	101	44.5	Residual blocks and more layers	76.4%
MobileNet V2	53	3.47	Inverted residual with linear bottleneck	72.1%

**TABLE 2.** Number of mislabels found in MNIST data. The five classifiers used here are: ResNet-50, VGG-16, MobileNet V2, Inception V3, and ResNet-101.

Voting method	Number of mislabels	% in total dataset
3/5 (Majority Filtering)	1825	3.04
4/5 (Majority Filtering)	751	1.25
5/5 (Consensus Filtering)	226	0.38

**TABLE 3.** Number of data available in each class. The nanopore experiment was done at 100 mV for three types of AAVs: single-stranded, double-stranded, and empty. The segment size shown in Fig. 7 was kept at 1 sec.

Class label	Number of data	Train set size (80%)	Test set size (20%)
Single	459	367	92
Double	181	145	36
Empty	120	96	24
**Total**	**760**	**608**	**152**

**TABLE 4.** Number of false positives (FP) and false negatives (FN) identified by different ensemble methods.

Number of classifiers	Voting method		Confidence threshold				
			0.9	0.8	0.7	0.6	0.5
**5**	**3/5**	**FP**	11	14	25	21	35
**5**	**3/5**	**FN**	3	1	1	1	0
**5**	**4/5**	**FP**	2	3	7	15	14
**5**	**4/5**	**FN**	5	5	4	2	2
**5**	**5/5**	**FP**	1	2	1	4	6
**5**	**5/5**	**FN**	13	14	9	6	5
**3**	**2/3**	**FP**	11	27	31	45	45
**3**	**2/3**	**FN**	4	4	1	3	0
**3**	**3/3**	**FP**	5	7	9	11	8
**3**	**3/3**	**FN**	10	4	5	4	3

**TABLE 5.** Prediction from individual classifier to identify the original label for a particular run of 5 classifiers with 4/5 voting and 0.80 threshold. 20 labels were modified randomly. Both true labels and modified labels are shown. A red-shaded cell indicates an incorrect prediction by the corresponding classifier. Here, S = Single, D = Double and E = Empty.

Data	Prediction					True label	Modified label
	*ResNet-50*	*VGG-16*	*Inception*	*EfficientNet*	*ResNet-101*		
1	S	S	S	S	S	**S**	**D**
2	D	D	D	D	D	**D**	**S**
3	S	S	S	S	S	**S**	**D**
4	S	S	S	S	S	**S**	**D**
5	S	S	S	S	S	**S**	**D**
6	S	S	S	S	S	**S**	**D**
7	S	S	S	S	S	**S**	**E**
8	D	D	S	D	D	**D**	**E**
9	D	D	S	D	S	**D**	**E**
10	S	S	S	S	S	**S**	**D**
11	S	S	S	S	S	**S**	**D**
12	S	S	S	S	S	**S**	**D**
13	D	D	E	D	D	**D**	**E**
14	S	S	S	S	S	**S**	**E**
15	E	E	E	E	E	**E**	**D**
16	S	S	D	S	S	**S**	**E**
17	S	S	S	S	S	**S**	**E**

**TABLE 6.** Prediction from individual classifier to predict the original label for a particular run of 3 classifiers with 2/3 voting and 0.50 threshold. 20 labels were modified randomly. Both true labels and modified labels are shown. A red-shaded cell indicates a wrong prediction by the corresponding classifier. Here, S = Single, D = Double and E = Empty.

Data	Prediction			True label	Modified label
	*ResNet-50*	*VGG-16*	*Inception*		
1	S	S	S	**S**	**D**
2	S	S	D	**S**	**D**
3	D	D	D	**D**	**S**
4	S	S	S	**S**	**D**
5	S	S	S	**S**	**D**
6	S	S	S	**S**	**D**
7	S	S	S	**S**	**D**
8	S	S	S	**S**	**E**
9	D	D	D	**D**	**E**
10	D	D	S	**D**	**E**
11	S	S	S	**S**	**D**
12	S	S	D	**S**	**D**
13	S	S	S	**S**	**D**
14	D	D	E	**D**	**E**
15	S	S	S	**S**	**E**
16	E	E	E	**E**	**D**
17	S	S	S	**S**	**E**
18	S	S	S	**S**	**E**

**TABLE 7.** Prediction from an individual classifier to identify the original label for a particular run of 5 classifiers with 4/5 voting. Here, Inception was replaced by MobileNet. All other conditions are the same as in Table 5. A red-shaded cell indicates a wrong prediction by the corresponding classifier. Here, S = Single, D = Double and E = Empty.

Data	Prediction					True label	Modified label
	*ResNet-50*	*VGG-16*	*MobileNet*	*EfficientNet*	*ResNet-101*		
1	S	S	S	S	S	**S**	**D**
2	D	D	D	D	D	**D**	**S**
3	S	S	S	S	S	**S**	**D**
4	S	S	S	S	E	**S**	**D**
5	S	S	S	S	S	**S**	**D**
6	S	S	S	S	S	**S**	**D**
7	S	S	S	S	S	**S**	**E**
8	D	D	D	D	D	**D**	**E**
9	S	S	S	S	S	**S**	**D**
10	S	S	S	S	S	**S**	**D**
11	S	S	S	S	S	**S**	**D**
12	D	D	D	D	D	**D**	**E**
13	S	S	S	S	S	**S**	**E**
14	E	E	E	E	E	**E**	**D**
15	S	S	S	S	S	**S**	**E**
16	S	S	S	S	S	**S**	**E**

**TABLE 8.** Prediction from an individual classifier to identify the original label for a particular run of 3 classifiers with 2/3 voting. Like Table 7, Inception was replaced by MobileNet. All other conditions are the same as in Table 6. A red-shaded cell indicates a wrong prediction by the corresponding classifier. Here, S = Single, D = Double and E = Empty.

Data	Prediction			True label	Modified label
	*ResNet-50*	*VGG-16*	*MobileNet*		
1	S	S	S	**S**	**D**
2	S	S	S	**S**	**D**
3	D	D	D	**D**	**S**
4	S	S	S	**S**	**D**
5	S	S	S	**S**	**D**
6	S	S	S	**S**	**D**
7	S	S	S	**S**	**D**
8	S	S	S	**S**	**E**
9	D	D	D	**D**	**E**
10	D	D	S	**D**	**E**
11	S	S	S	**S**	**D**
12	S	S	S	**S**	**D**
13	S	S	S	**S**	**D**
14	D	D	D	**D**	**E**
15	S	S	S	**S**	**E**
16	E	E	E	**E**	**D**
17	S	S	S	**S**	**E**
18	S	S	S	**S**	**E**

**TABLE 9.** Total runtime and its breakdown for different components.

Case	Total time taken (sec)	Breakdown of total time			
		Component	Classifiers	Time (sec)	% of total time
**Five classifiers ensemble**	**34673.10**	1. Training	a) ResNet-50	5044.01	14.55
			b) VGG-16	13945.49	40.22
			c) Inception V3	2986.24	8.61
			d) EfficientNet	2354.34	6.79
			e) ResNet-101	9982.72	28.79
			**Total**	**34312.80**	**98.96**
		2. Other (preprocessing, model initialization, prediction)		360.30	1.04
**Three classifiers ensemble**	**21148.88**	1. Training	a) ResNet-50	4874.38	23.05
			b) VGG-16	13304.92	62.91
			c) Inception V3	2796.27	13.22
			**Total**	**20975.56**	**99.18**
		2. Other (preprocessing, model initialization, prediction)		173.32	0.82

**TABLE 10.** Detailed prediction for a balanced dataset in a 2/3 voting case. 20 labels were modified randomly. Both true labels and modified labels are shown. A red cell indicates a wrong prediction by the classifier. Here, S = Single, D = Double and E = Empty.

Data	Prediction			True label	Modified label
	*ResNet-50*	*VGG-16*	*MobileNet*		
1	E	E	E	**E**	**D**
2	E	E	E	**E**	**D**
3	S	E	D	**E**	**S**
4	D	D	D	**D**	**E**
5	E	E	E	**E**	**D**
6	E	E	E	**E**	**D**
7	E	E	E	**E**	**D**
8	S	S	D	**S**	**D**
9	S	S	S	**S**	**E**
10	S	S	S	**S**	**D**
11	D	D	D	**D**	**E**
12	D	D	E	**D**	**E**
13	D	E	E	**E**	**D**
14	D	D	D	**D**	**E**
15	S	S	S	**S**	**D**
16	E	D	E	**E**	**D**
17	D	D	D	**D**	**S**
18	S	S	S	**S**	**D**
19	S	S	D	**S**	**E**
20	D	S	S	**S**	**E**

**TABLE 11.** Comparison of results between balanced and unbalanced datasets for the same condition: 3 classifier ensemble (ResNet-50, VGG-16, and MobileNet), 2/3 voting.

Dataset	Dataset size	Mislabel		
		Injected	Identified	Missed
Unbalanced	760	20	18	2
Balanced	360	20	20	0

## Data Availability

Data will be made available on request.

## APPENDIX A

### CLUSTERING RESULTS USING KNN AND K-MEANS

**k-Nearest Neighbor (KNN)** with k = 5 was implemented for a total dataset of 760 (25% for testing). An accuracy of 70.52% was achieved for the test dataset. The confusion matrix is shown in Fig. 13.

Similarly, clustering was performed with **K-means,** and accuracy was even lower (49.86%). The corresponding confusion matrix is presented in Fig. 14.

The full dataset was visualized after dimensionality reduction via principal component analysis (PCA) with 2 principal components. The result is shown in Fig. 15.

The data visualization in Fig. 15 indicates that the three classes are not separated into three distinct zones, and there are substantial overlaps among these zones. As a result, we get lower accuracies in classical clustering techniques.

## APPENDIX B

### NEAR-HOMOGENEOUS ENSEMBLE

Three shallow neural network-based classifiers are developed using the same CNN backbone, but with variable ANN (fully connected) layers added on top. In other words, only changes were made to the final fully connected layers in these near-homogeneous models.

The model was trained on a similar set of AAV data described in the main body of the paper. Although the three-classifier configuration required only 64 minutes of runtime, its performance was significantly inferior to that of the pretrained models. The customized CNN variants did not achieve comparable accuracy, resulting in a high number of false positives—most notably, 166 out of 760 samples.

## APPENDIX C

### DivideMix FOR MISLABEL IDENTIFICATION IN AAV DATASET

The workflow used for DivideMix is shown in Fig. 17, which begins with AAV dataset preprocessing, including image resizing/normalization and controlled mislabel injection at predefined index positions to simulate label noise. Two independent networks with identical backbone architectures (ResNet-50) are initialized using the Adam optimizer with a learning rate $l_{r}$ and weight decay parameter λ. The models are first trained for a short warmup phase $\left(E_{warm}=5\right)$ using standard cross-entropy loss on the noisy labels to stabilize feature representations and enable meaningful loss separation. After the warmup, the main DivideMix training loop is executed. At each epoch, per-sample losses from both networks are computed and used to fit a two-component Gaussian mixture model (GMM), which estimates the probability of each sample being clean. Based on these probabilities, the dataset is dynamically partitioned into labeled (clean) and unlabeled (noisy) subsets. A co-divide strategy is employed, where each network is trained using the partition determined by the other network, thereby reducing confirmation bias. During the semi-supervised learning stage, MixMatch is applied to refine labels for the labeled subset and generate reliable pseudo-labels for the unlabeled subset, followed by a regularization. Training continues until validation accuracy no longer improves for a predefined patience threshold, at which point early stopping is triggered and the best-performing model weights are restored. The resulting labeled/unlabeled (L/U) split statistics and final classification accuracy are then reported. For instance, for our AAV dataset, DivideMix reported 623 “labeled” and 137 “unlabeled” samples for only twenty mislabeled samples. This indicates that DivideMix aggressively treats many clean samples as noisy under low-noise conditions. This behavior is expected, as DivideMix is designed for scenarios with moderate-to-high noise ratios and large-scale datasets, where noise modeling via mixture estimation is statistically meaningful. In contrast, our problem setting involves a small number of subtle mislabels in a relatively clean biomedical dataset, where overly aggressive filtering can discard valuable data.
